# Core beliefs in psychosis: a systematic review and meta-analysis

**DOI:** 10.1038/s41537-025-00577-2

**Published:** 2025-03-06

**Authors:** A. Jorovat, R. Twumasi, A. Mechelli, A. Georgiades

**Affiliations:** 1 Department of Psychosis Studies, Institute of Psychiatry, Psychology, and Neuroscience (IoPPN), King’s College London, London, UK; 2 Brent Early Intervention Service, CNWL, NHS Foundation Trust, 27-29 Fairlight Avenue, London, NW10 8AL UK

**Keywords:** Diseases, Psychosis

## Abstract

Increasing interest is growing for the identification of psychological mechanisms to account for the influence of trauma on psychosis, with core beliefs being proposed as a putative mediator to account for this relationship. A systematic review (*n* = 79 studies) was conducted to summarise the existing evidence base regarding the role of core beliefs/schemas in psychosis, Clinical High-Risk (CHR), and non-clinical samples with Psychotic-Like Experiences (PLEs). Compared to Healthy Controls (HCs), individuals with psychosis experiencing Auditory Hallucinations or Persecutory Delusions had significantly higher scores for negative self and negative other-beliefs and significantly lower scores for positive self and positive other-beliefs. This pattern of core beliefs was also observed for CHR individuals. In contrast, the core belief profile for grandiose delusions was in the opposite direction: higher positive self and positive other-beliefs and lower negative self-beliefs. In non-clinical samples, several factors mediated the relationship between Traumatic Life Events (TLEs) and PLEs, such as greater perceived stress, dissociation, external locus of control, and negative self and negative other-beliefs. Compared to HCs, meta-analyses revealed statistically significant large effects for negative self and negative other-beliefs in Schizophrenia. In CHR, statistically significant large and moderate effects were found for negative self and negative other-beliefs, respectively, along with a moderate negative effect for positive self-beliefs. Core beliefs were found to play a significant role in the development and maintenance of positive symptoms of psychosis. The development of psychosocial interventions that explicitly target negative self and other-beliefs, whilst also enhancing positive self-beliefs are warranted and would innovate CBTp practices.

## Introduction

Trauma has repeatedly been implicated in the onset and exacerbation of psychosis^1,2^, with 89% of individuals with a First Episode Psychosis (FEP) reporting one or more adversities compared to 37% of controls^3^. Specifically, childhood/adolescent sexual, physical, and emotional abuse, physical/emotional neglect, separation, and institutionalisation were 4–17 times higher for the FEP group. Moreover, for each additional adversity, the risk of psychosis increased 2.5 times, indicating a dose-response effect of adversity exposure on psychosis risk^3^. Increasing interest is growing for the identification of psychological mechanisms to account for the influence of trauma on psychosis^4,5^, with core beliefs being proposed as a putative mediator to account for this relationship^5^.

Core beliefs have been defined as fundamental, inflexible, absolute, and generalised beliefs an individual holds about themselves, others, and the world^6^. The term core belief refers to a relatively enduring cognitive template for organising and processing external stimuli^7^ and is often used interchangeably with the term schema^8^. Schemas have been defined as stable and trait-like vulnerability factors^9^ that are comprised of memories, cognitions, emotions, and bodily sensations regarding oneself and one’s relationship with others, originating from childhood needs^10^. Core beliefs have therefore been conceptualised as the cognitive component of an underlying schematic theme^7,11^. Early life experiences have thus been considered influential in the formation of core beliefs, leading an individual to view themselves as a failure, inferior, weak/vulnerable, worthless, and/or unlovable/unlikeable, others as untrustworthy, uncaring, unreliable, and/or rejecting, and the world as dangerous, unpredictable, and/or isolating^11,12^. Indeed, negative core beliefs have been associated with positive symptoms of psychosis such as Auditory Hallucinations (AH)^13,14^, Persecutory Delusions (PD)^15–17^, and Clinical High-Risk (CHR) status^18,19^. In terms of AHs, significant associations have been found between negative self and negative other-beliefs with AH expression, persecutory beliefs about voices, and voice related distress^20,21^. Additionally, Persecutory Delusions have been found to positively correlate with negative self-beliefs and to negatively correlate with positive self-beliefs^22^. Moreover, negative self-beliefs, depression, and anxiety have been found to significantly predict an increased chance of PD^23^. However, compared to persecutory delusions, grandiose delusions were associated with less negative self-beliefs coupled with higher positive self and positive other-beliefs^23^, highlighting symptom-specific core belief profiles across psychosis presentations. In terms of schemas and suicidal risk in SZ, the Emotional Deprivation schema has been found to correlate with a 1.56 increased risk of lifetime suicide attempts and has been significantly associated with positive symptoms, negative symptoms, and depression^24^. This highlights the importance of identifying schemas and their potential involvement in the exacerbation of suicidal risk and self-harm in psychosis. Moreover, higher negative self and negative other-beliefs^17,25–27^, as well as lower positive self and positive other-beliefs^25,27^ have been significantly correlated with the severity of suicidal ideation in SZ, highlighting their clinical significance within psychosis samples.

In terms of trauma and core belief formation in SZ, childhood interpersonal trauma positively and significantly correlated with disorganised attachment, which in turn was associated with negative self and negative other-beliefs, and paranoia^15^. Disorganised attachment and negative other-beliefs fully mediated the relationship between trauma and paranoia^15^. Negative other-beliefs were also found to partially mediate the association between disorganised attachment and paranoia^15^. Moreover, negative other-beliefs were found to mediate the relationship between emotional abuse and rumination in SZ^28^. These findings implicate the role of trauma and disorganised attachment in the development of core beliefs, and their associations with symptoms of psychosis.

In terms of trauma, CHR individuals have been found to experience significantly more types of traumas (emotional/physical/sexual abuse, emotional/physical neglect), and bullying compared to healthy controls^18,29^. In terms of core beliefs in CHR, negative self and negative other-beliefs have been found to positively correlate with total trauma and trauma subtypes^29^. Moreover, bullying was significantly positively correlated with negative self^30^ and negative other-beliefs^19,30^ and Distressing Unusual Experiences (UEDs)^30^. Interestingly, negative self and negative other-beliefs were found to significantly mediate the relationship between bullying and UEDs^30^. These findings highlight the high prevalence of trauma and bullying in CHR, the presence of negative self and negative other core beliefs among CHR, and the emerging evidence for their association with unusual experiences.

Negative beliefs about self and others have also been found to play a mediating role between childhood trauma/social adversity and paranoia in SZ^15^ and between Traumatic Life Events (TLEs) and Psychotic-Like Experiences (PLEs) in non-clinical samples^31^. Conversely, positive self-beliefs have been associated with recovery in psychosis^32^, which highlights important clinical implications. It has therefore been posited that core beliefs may play a critical role in psychosis symptom emergence in CHR and FEP in response to psychosocial stress and may be an important clinical intervention target^33,34^.

Therefore, a systematic review and meta-analysis summarising the role of core beliefs in psychosis symptom expression may help contribute to formulation development and targeted interventions in Cognitive Behavioural Therapy for Psychosis (CBTp).

### Aims of the study

The aim of this systematic review and meta-analysis is to provide a comprehensive summary of the existing literature and to quantify the effects of core beliefs in psychosis and CHR, with the view to proposing cognitive models to outline the influence of core beliefs in psychosis onset and relapse. The role of core beliefs in relation to suicidal risk in psychosis will also be explored. Additionally, as early life experiences and trauma events are hypothesised to contribute to the formation of core beliefs, the association between specific trauma subtypes (e.g. sexual, physical, emotional abuse and physical and emotional neglect) in relation to core beliefs/schemas in psychosis will also be outlined.

## Methods

This review was conducted in accordance with the Preferred Reporting Items for Systematic Reviews and Meta-Analyses (PRISMA) guidelines^35^. Methods and inclusion criteria were specified in advance and documented in a protocol registered with the PROSPERO International Prospective Register of Systematic Reviews (registration number: CRD42024552881).

### Search strategy

A systematic database search of MEDLINE (including PubMed), EMBASE, Global Health and APA PsycINFO was conducted using an OVID search tool from database conception to 30th June 2024. The following search strings were used: Psychosis OR Psychotic OR Schizophreni* **AND** Core belief* OR Schema* **AND** Delusion* OR Hallucination* OR Positive symptom* OR Negative symptom* OR Psychotic symptom* OR paranoi*.

### Study selection

**Fig. 1:**
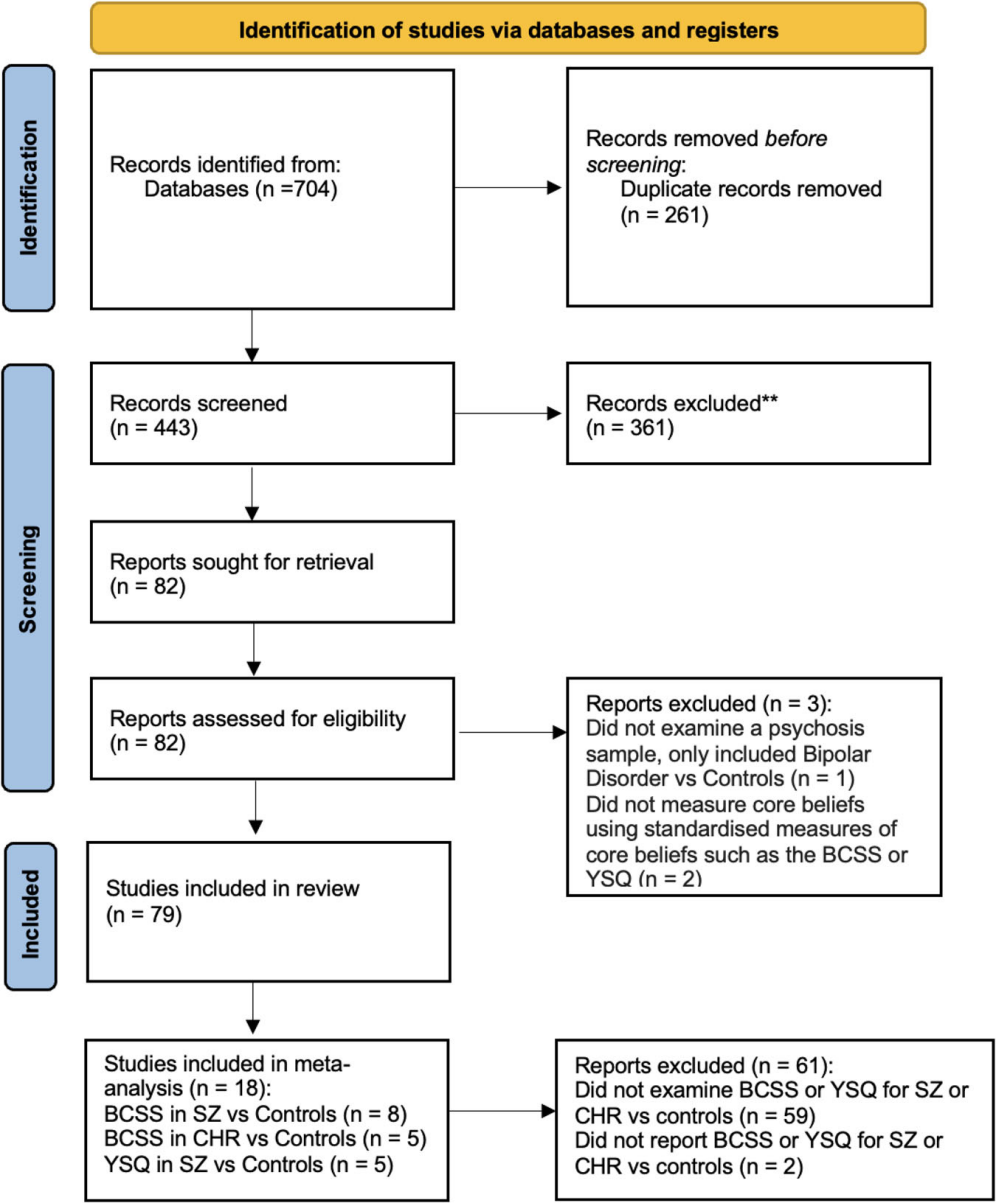
PRISMA 2020 flow diagram.

Studies, regardless of their design, publication date or length of follow-up, were considered. Eligible studies needed to (a) explore the role of core beliefs and/or schemas in psychotic disorders (Schizophrenia, First Episode Psychosis [clinical status will be reported according to how the included studies have reported their clinical samples]), CHR, and/or non-clinical samples with PLEs, and/or (b) explore interventions that target core beliefs and/or schemas in psychosis, CHR, and/or non-clinical samples with PLEs. Studies were eligible for inclusion if they were published in peer-reviewed journals, written in English, and included participants with a psychosis disorder diagnosed using a reliable psychometric tool (e.g. Diagnostic and Statistical Manual of Mental Disorders, fifth edition, or DSM-5^36^; International Classification of Diseases, eleventh edition, or ICD-11^37^). The study selection process is summarised in the PRISMA flow diagram (see Fig. 1).

### Exclusion criteria

Studies written in languages other than English, unpublished or grey literature, conference abstracts, book chapters, meta-analyses, reviews, and case studies were excluded from this review. Studies were also excluded if patients had drug-induced psychosis or psychosis due to organic causes. Lastly, studies were excluded if they did not investigate an association between core beliefs/schemas and psychosis experiences.

### Data extraction process

Relevant literature was de-duplicated and imported into Microsoft Excel. An Excel spreadsheet was created to record the characteristics of studies. The extracted data included different variables: author (name/year), country and type of study, sample size and setting, mean age (SD), questionnaires/diagnostic tools, main findings and clinical implications. The screening process was conducted independently by one reviewer (A.J) and was subsequently cross-checked by a second reviewer (A.G).

### Meta-analysis

Studies were included in the meta-analysis if they reported the means and standard deviations for a clinical group (SZ/CHR) and healthy controls on either the BCSS or the YSQ. All corresponding authors of studies included in the systematic review were contacted to ask for raw data, three authors sent data for analysis. For the BCSS, only 12 studies met inclusion criteria (*k* = 8 SZ; *k* = 5 CHR), and for the YSQ, only 5 studies (all SZ) met inclusion criteria.

### Statistical analysis

All analyses were conducted using R (version 4.4.2) with the metafor package^38^. Standardised mean differences (SMD) were calculated using the escalc function as units of Cohen’s *d*. Random-effects models were fitted using restricted maximum likelihood estimation (REML) with Knapp-Hartung adjustments to provide a more conservative inference, particularly given the relatively small number of included studies. Studies were weighted by the inverse-variance method. Between-study heterogeneity was assessed using Cochran’s *Q* test and *I*² statistics, where *I*² values of 25%, 50%, and 75% indicate low, moderate, and high heterogeneity, respectively. The *τ*² statistic was computed to estimate the between-study variance. For each analysis, 95% confidence intervals were calculated for the pooled effect sizes. To account for the impact of heterogeneity on future studies, we computed 95% prediction intervals^39^. Unlike confidence intervals which estimate the uncertainty around the mean effect, prediction intervals provide the range within which the true effect size would be expected to fall in 95% of similar future studies, taking into account both sampling error and between-study heterogeneity. This approach offers a more comprehensive understanding of effect size variability across different research contexts and populations. Given the limited number of studies (*k* < 10 in all models), publication bias assessment using funnel plots, Egger’s regression test, and trim-and-fill analyses was deemed inappropriate due to insufficient power, following recommendations by Dalton et al.^40^ which suggests a rule of thumb of at least 10 studies in each model for estimating publication bias. This limitation is acknowledged as a potential source of bias in our findings. Meta-regression was not conducted due to *k* < 10 and the risk of overfitting^41,42^.

**Fig. 2:**
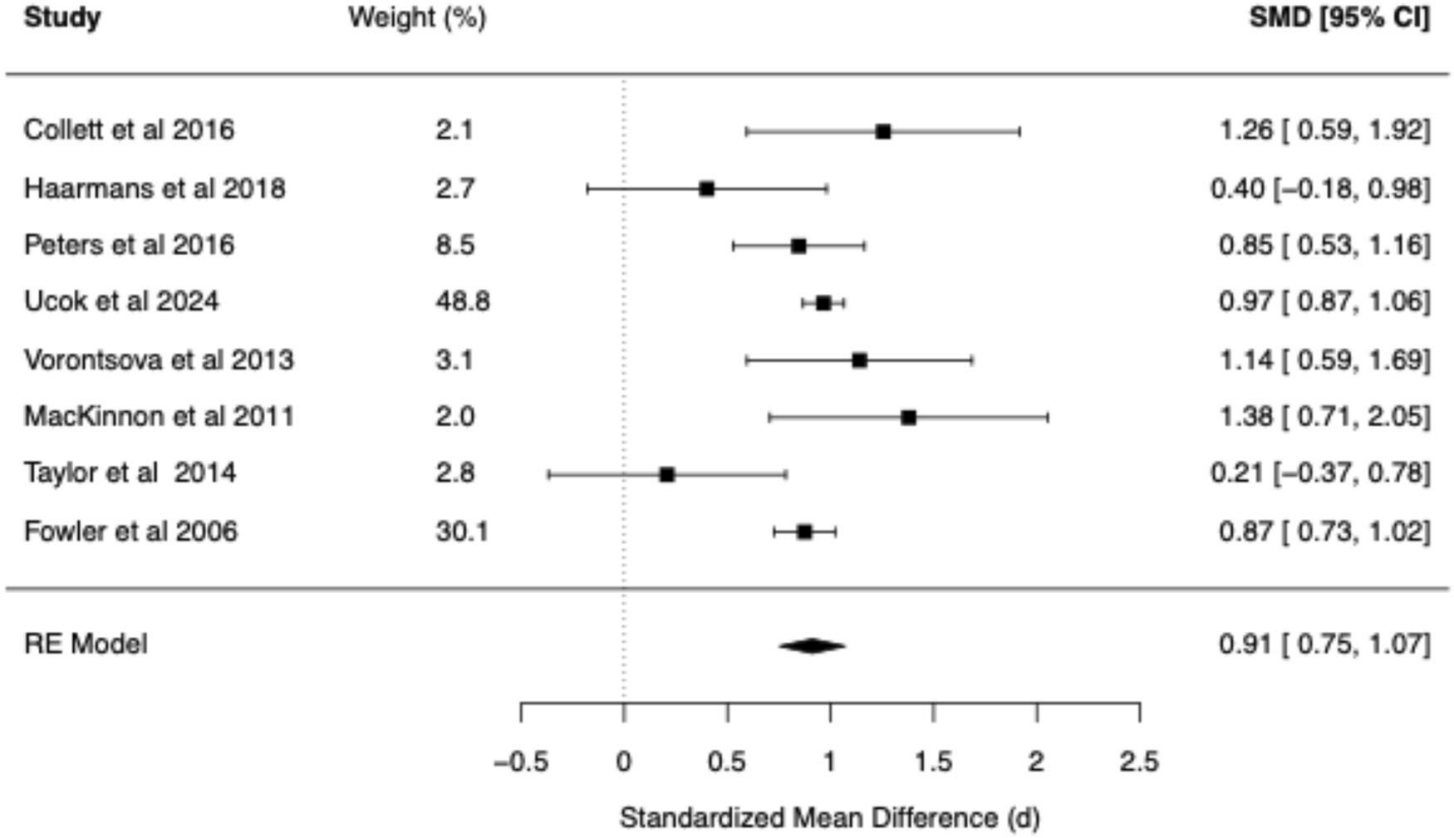
Forest plot of BCSS negative self beliefs in schizophrenia vs. healthy controls.

**Fig. 3:**
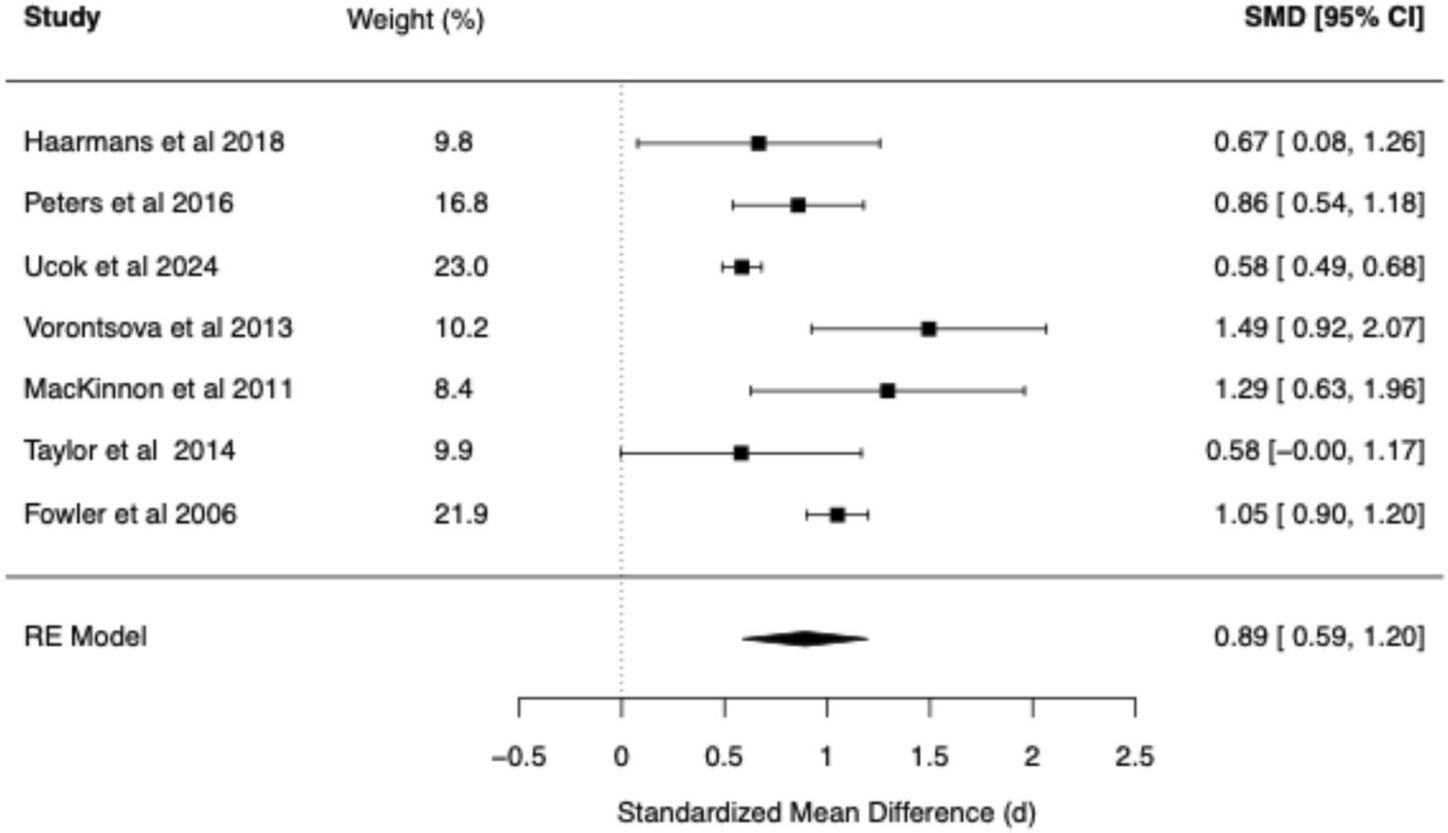
Forest plot of BCSS negative other beliefs in schizophrenia vs. healthy controls.

**Fig. 4:**
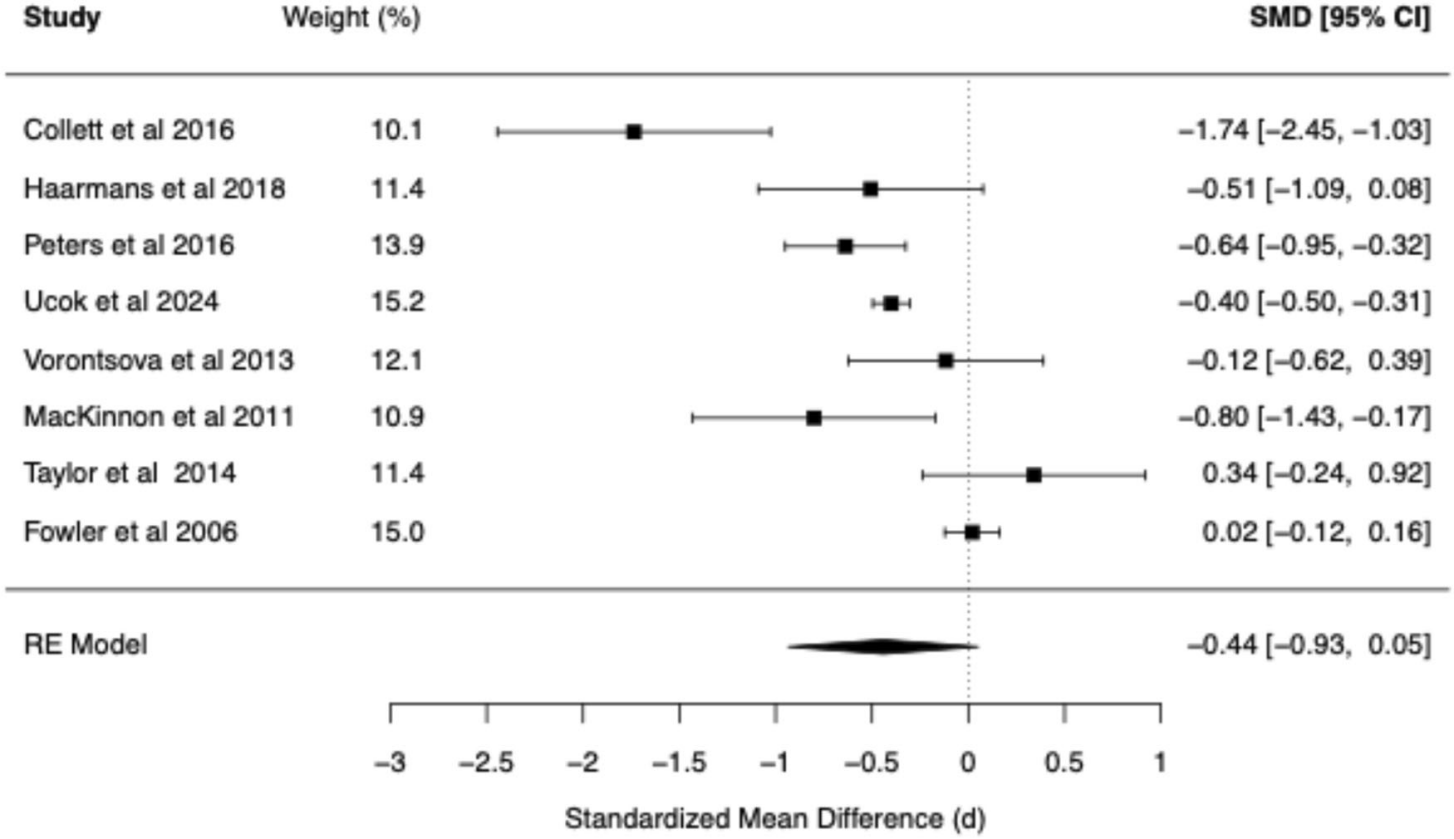
Forest plot of BCSS positive self beliefs in schizophrenia vs. healthy controls.

**Fig. 5:**
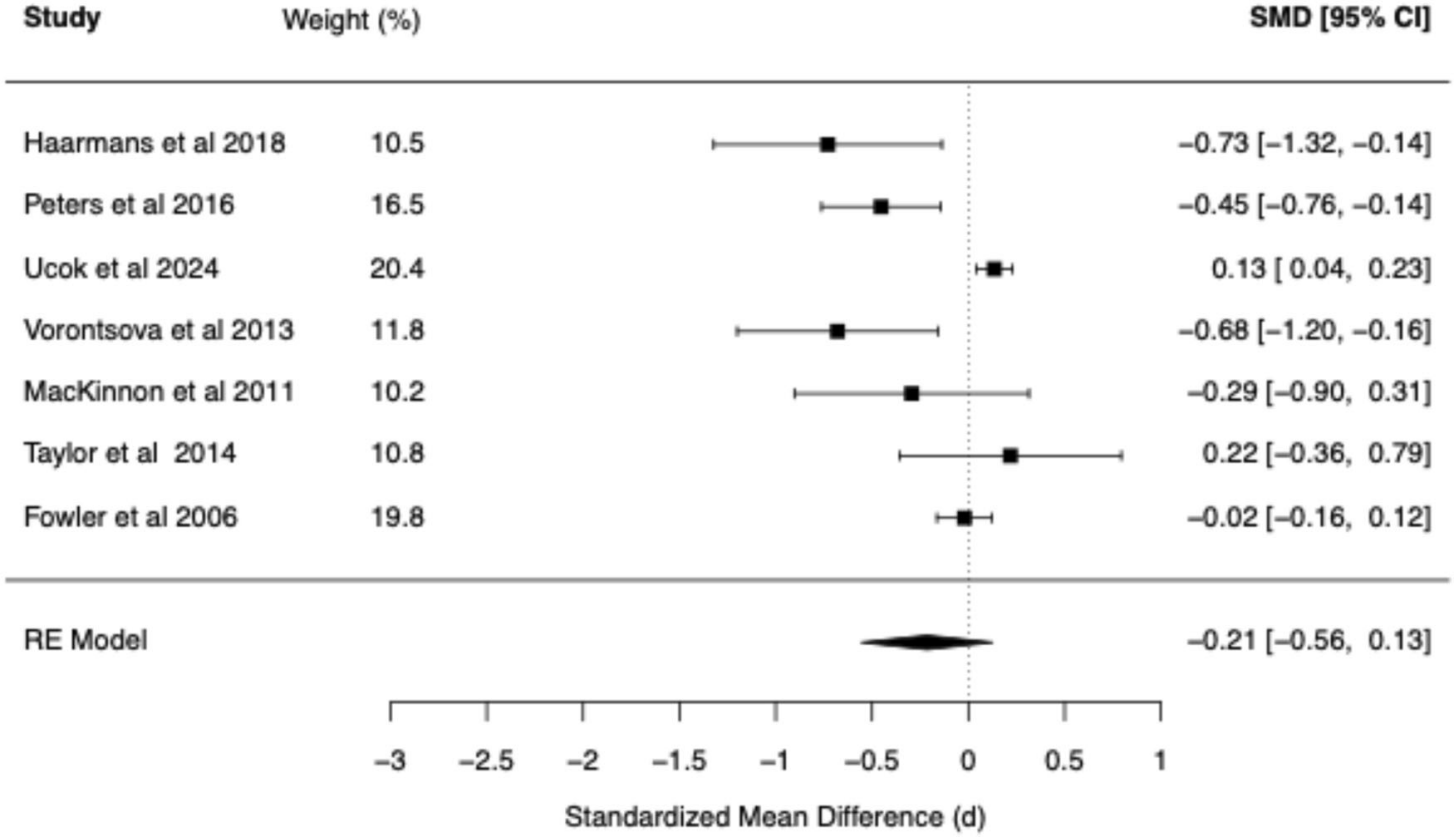
Forest plot of BCSS positive other beliefs in schizophrenia vs. healthy controls.

**Fig. 6:**
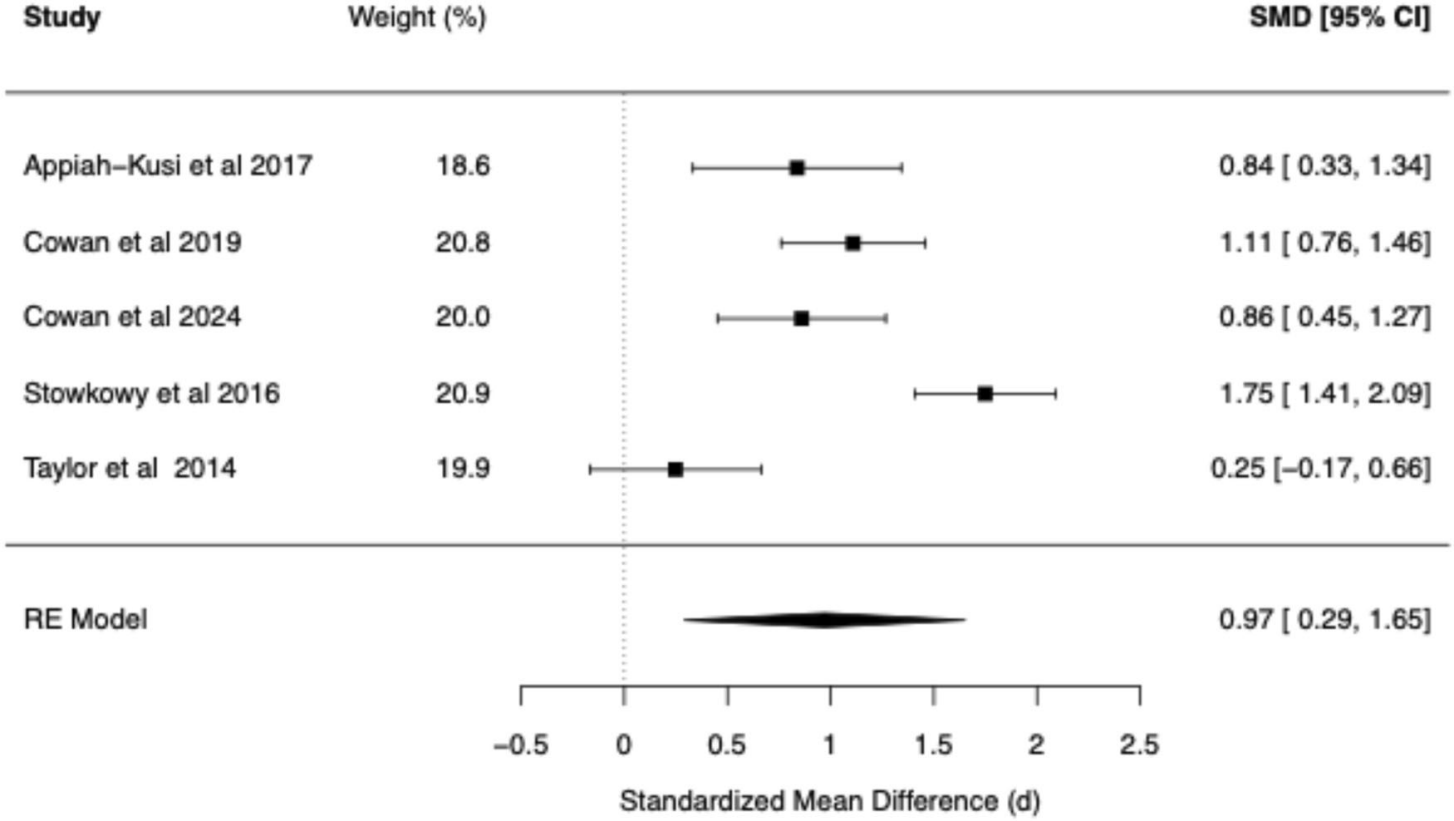
Forest plot of BCSS negative self beliefs in clinical high risk vs. healthy controls.

**Fig. 7:**
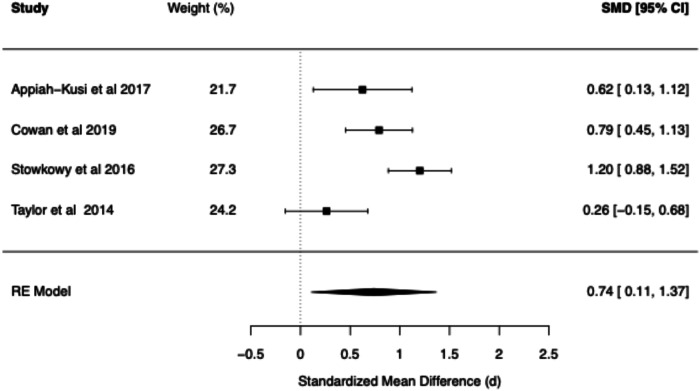
Forest plot of BCSS negative other beliefs in clinical high risk vs. healthy controls.

**Fig. 8:**
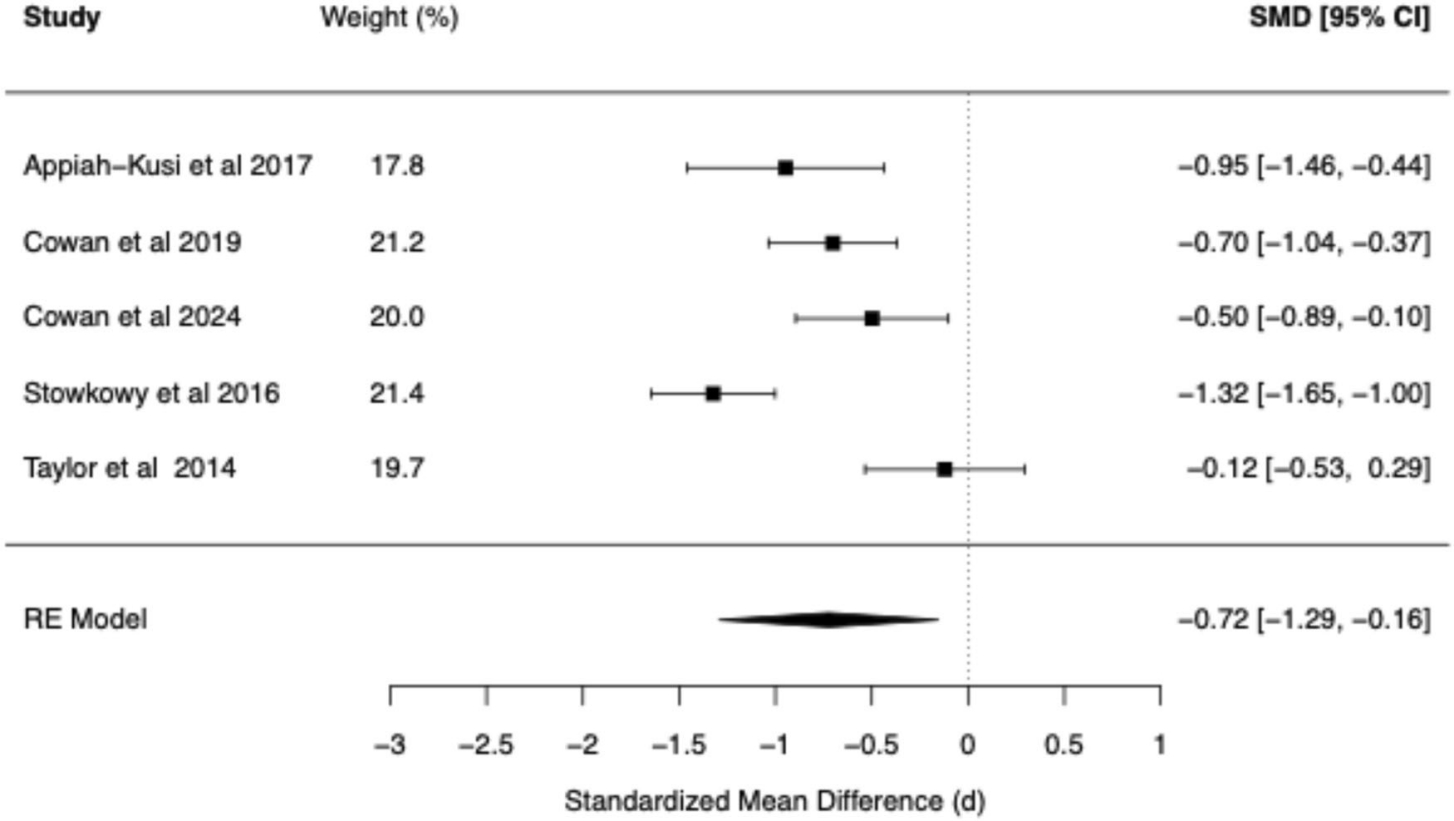
Forest plot of BCSS positive self beliefs in clinical high risk vs. healthy controls.

**Fig. 9:**
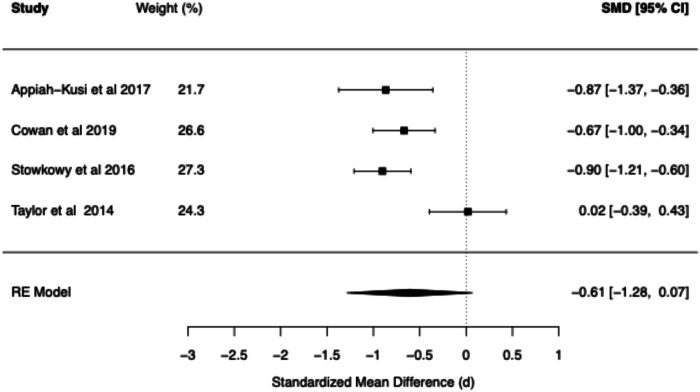
Forest plot of BCSS positive other beliefs in clinical high risk vs. healthy controls.

The meta-analytic findings for four core belief dimensions of the BCSS (negative self, negative other, positive self, positive other) are presented for SZ vs. Controls (see Table 1 and Figs. 2–5) and for CHR vs. Controls (see Table 2 and Figs. 6–9). The meta-analytic findings for 15 schemas of the YSQ are presented for SZ vs. Controls (see Table 3). Forest plots were generated for the BCSS analyses, displaying individual study effect sizes, weights, and pooled estimates with their respective confidence intervals.

**Table 1:** Summary of BCSS Meta-Analytic models—schizophrenia vs. healthy controls.

Core belief	Pooled RE model SMD (*d*)	95% confidence interval	*Q* statistic	Cochran’s *Q* test *p* value	*I*² (%)	*τ*²	95% prediction interval
Negative self	0.91	[0.75, 1.07]	13.76	0.06	11.51	0.00	[0.71, 1.11]
Negative other	0.89	[0.59, 1.20]	36.63	<0.01	79.16	0.06	[0.21, 1.58]
Positive self	−0.44	[−0.93, 0.05]	52.01	<0.01	93.51	0.25	[−1.73, 0.84]
Positive other	−0.21	[−0.56, 0.13]	29.52	<0.01	85.75	0.09	[−1.03, 0.60]

**Table 2:** Summary of BCSS Meta-Analytic models—clinical high risk vs. healthy controls.

Core belief	Pooled RE model SMD (*d*)	95% confidence interval	*Q* statistic	Cochran’s *Q* test *p* value	*I*² (%)	*τ*²	95% prediction interval
Negative self	0.97	[0.29, 1.65]	32.18	<0.01	86.62	0.26	[−0.61, 2.55]
Negative other	0.74	[0.11, 1.37]	13.08	<0.01	76.25	0.12	[−0.54, 2.01]
Positive self	−0.72	[−1.29, −0.16]	23.27	<0.01	81.80	0.17	[−2.01, 0.56]
Positive other	−0.61	[−1.28, 0.07]	13.35	<0.01	78.80	0.14	[−1.97, 0.75]

**Table 3:** Summary of YSQ Meta-Analytic models.

Schema	Pooled RE model SMD (*d*)	95% confidence interval	*Q* statistic	Cochran’s *Q* test *p* value	*I*² (%)	*τ*²	95% prediction interval
Abandonment	0.87	[0.57, 1.17]	6.50	0.16	39.63	0.02	[0.34, 1.40]
Mistrust/abuse	1.27	[0.58, 1.96]	28.39	<0.00	86.71	0.27	[−0.33, 2.87]
Emotional deprivation	1.14	[0.70, 1.59]	14.00	0.01	69.62	0.09	[0.20, 2.09]
Defectiveness/shame	0.90	[0.51, 1.29]	11.40	0.02	63.46	0.06	[0.10, 1.71]
Social Isolation	1.16	[0.30, 2.03]	26.72	<0.00	90.91	0.39	[−0.77, 3.10]
Dependence/incompetence	0.97	[0.43, 1.52]	17.56	<0.00	78.59	0.14	[−0.20, 2.14}
Vulnerability to harm	0.98	[0.39, 1.57]	15.85	<0.00	79.96	0.15	[−0.25, 2.21]
Enmeshment	0.94	[0.71, 1.17]	3.83	0.43	0.01	0.00	[0.71, 1.17]
Failure	0.89	[0.46, 1.32]	13.54	0.01	69.26	0.08	[−0.02, 1.80]
Entitlement	0.98	[0.51, 1.45]	8.16	0.04	61.84	0.06	[0.08, 1.88]
Insufficient self-control	0.85	[0.49, 1.22]	8.03	0.09	47.55	0.03	[0.23, 1.48]
Subjugation	0.86	[0.50, 1.23]	9.15	0.06	56.63	0.05	[0.15, 1.58]
Self-sacrifice	0.50	[0.27, 0.72]	3.99	0.41	0.02	0.00	[0.27, 0.72]
Emotional inhibition	0.76	[0.39, 1.14]	11.29	0.02	62.42	0.06	[−0.02, 1.54]
Unrelenting standards	0.57	[−0.01, 1.15]	25.07	<0.00	83.73	0.19	[−0.76, 1.90]

Full R code to reproduce our analysis and extracted data is publicly available here: https://github.com/ricardotwumasi/psychosis-core-beliefs ensuring reproducibility and transparency of our approach.

### Quality appraisal

The methodological quality of the included studies was assessed using the Effective Public Health Practice Project (EPHPP)^43^ for quantitative studies (see Appendix 1) and the Joanna Briggs Institute Critical Appraisal Checklist for Qualitative Research (Joanna Briggs Institute^44^) for qualitative studies (see Appendix 2).

## Results

Of the 704 papers initially identified through database screening, a total of 79 studies were included in this review. Appendix 3–5 summarise the characteristics and main findings of all included studies.

### Risk of bias and certainty assessment

According to the EPHPP tool, the global quality ratings for the included studies were as follows: ‘strong’ (*n* = 45), ‘moderate’ (*n* = 30), and ‘weak’ (*n* = 3) (see Appendix 1). Additionally, the one included qualitative study scored a global rating of ‘strong’ according to the JBI tool (see Appendix 2). This suggests that the majority of the studies included in the present review included low risk of bias studies.

### Psychosis

#### Core beliefs and schemas

Out of the 43 studies examining psychosis populations, only five examined Early Maladaptive Schemas (EMS) using the YSQ in SZ^16,45–48^. Compared to HCs, SZ patients demonstrated significantly higher scores on all schemas^47,48^, except for unrelenting standards^16,45,46^, emotional inhibition^46^, and self-sacrifice^16,45^. According to one study, only six schemas remained statistically significant after controlling for depression, namely emotional deprivation, social isolation, defectiveness/shame, enmeshment, failure, and subjugation^45^. In two studies, schemas significantly predicted positive, but not negative symptoms of psychosis^45,46^. Specifically, enmeshment, failure, subjugation, mistrust/abuse, social isolation, and vulnerability to harm schemas were significantly associated with positive symptoms^45^. After controlling for depression, only the mistrust/abuse schema was found to significantly predict positive symptoms, explaining 12.4–22% of the variance^45,47^. In contrast, after controlling for depression, only the social isolation schema was found to significantly predict negative symptoms, explaining 27% of the variance^47^. In an experimental study examining EMS and stress induction using the Cyberball paradigm in PDs and HCs, the PD group responded with a stronger increase in paranoia and demonstrated a significantly higher total EMS score compared to HCs^16^. Higher increases in paranoia following social stress were accounted for by higher EMS total scores with the Defectiveness/Shame and Enmeshment/Underdeveloped Self schemas being associated with stress related symptom increases in PD^16^.

Compared to HCs, SZ patients scored significantly higher on negative self^17,28,49–54^ and negative other-beliefs^28,49–54^ and significantly lower on positive self^17,51–54^ and positive other-beliefs^49,51,52^. These findings highlight the schematic and core belief profile of SZ.

#### Trauma

Six studies explored the associations between trauma, core beliefs, and SZ, focusing on how childhood trauma influences core beliefs and symptomatology^15,28,54–56^. Firstly, a high prevalence of childhood interpersonal trauma was found, with 89% reporting at least one trauma^15^. Another study found that 74.1% of SZ patients reported victimisation trauma and 21.5% met symptomatic criteria for PTSD^56^. All types of trauma subtypes were significantly more prevalent in SZ compared to their Siblings (SIB) and HCs: physical abuse (63% SZ vs. 17% SIB, 11.1% HC), sexual abuse (32.6% SZ vs. 15.9% SIB, 9.8% HC), emotional abuse (43.3% SZ vs. 22.1% SIB, 14.8% HC), physical neglect (58.2% SZ vs. 44% SIB, 39.5% HC), and emotional neglect (55.2% SZ vs. 42.9% SIB, 34% HC)^54^.

Childhood interpersonal trauma was positively and significantly associated with disorganised attachment, which in turn was associated with negative self and negative other-beliefs, and paranoia^15^. After controlling for depression, hallucinations, and age, disorganised attachment and negative other-beliefs fully mediated the relationship between trauma and paranoia^15^. Negative other-beliefs also partially mediated the association between disorganised attachment and paranoia^15^. Moreover, negative other-beliefs mediated the relationship between emotional abuse and rumination in SZ^28^. Emotional abuse/neglect was significantly associated with PDs and delusions of influence, both of which were mediated by anxiety^55^. Childhood sexual abuse was significantly associated with AHs, which was mediated by post-traumatic avoidance, numbing, and hyperarousal^56^. Childhood emotional abuse was significantly associated with PDs and referential delusions, with the former being mediated by negative other-beliefs^56^. Negative self-beliefs mediated the relationship between emotional abuse and thought withdrawal and broadcast, with 33.9% of the variance in these symptoms explained by negative self-beliefs^54^. Negative self-beliefs also mediated the relationship between sexual abuse and PDs^54^. These findings signify the high prevalence of trauma in psychosis, along with the mediating role of negative self and other-beliefs for selected trauma-symptom associations.

#### Auditory hallucinations

Ten studies investigated AHs and core beliefs in SZ and FEP, and all found significant associations between negative self and/or negative other-beliefs with AH expression^13,14,20–22,33,49,57–59^. Compared to HCs, SZ and FEP experiencing AHs had significantly higher scores for negative self and negative other-beliefs^49,58^ and significantly lower scores for positive other-beliefs^49^. Additionally, negative self and negative other-beliefs significantly positively correlated with persecutory beliefs about the voices^20^, and the amount and intensity of voice-related distress^20,21^. Specifically, negative self-beliefs significantly positively correlated with beliefs about voices regarding malevolence, omnipotence, meta-physical beliefs, loss of control, and total negative AH content, and negatively correlated with benevolence^14,57,59^. Negative other-beliefs significantly positively correlated with omnipotence and loss of control^57^. Positive self-beliefs significantly negatively correlated with malevolence, omnipotence, meta-physical beliefs, and loss of control and significantly positively correlated with benevolence^57^. After controlling for negative voice content, negative self and negative other-beliefs significantly predicted between 9% and 35% of the variance in the six beliefs about voices (i.e. malevolence, benevolence, omnipotence, meta-physical, loss of control, and positive beliefs), with negative self-beliefs being the strongest predictors^13^. Moreover, a qualitative investigation found that negative self-beliefs contributed to psychosis symptoms, such as AHs, and the voices in turn, often reinforced negative self-beliefs^33^.

#### Persecutory delusions

Nine studies examined core beliefs in PD, and all found significant associations between negative self and/or negative other-beliefs with persecutory beliefs^20,22,23,50,54,56,60–62^. In SZ, paranoia significantly positively correlated with negative self-beliefs, and significantly negatively correlated with positive self-beliefs^22^. Higher levels of state paranoia were also significantly positively correlated with negative self-beliefs^60^. Moreover, negative self and negative other-beliefs significantly positively correlated with persecutory beliefs^20^. A Persecutory Only group demonstrated higher negative self and negative other-beliefs and lower positive self and positive other-beliefs compared to the Grandiose Only, Persecutory and Grandiose, and Neither Persecutory or Grandiose groups^23^. The Grandiose Only group had the highest positive self and positive other-beliefs and the lowest negative self-beliefs^23^. Negative self-beliefs, depression and anxiety predicted a significantly increased chance of PD^23^. Conversely, grandiose delusions were characterised by less negative self-beliefs and lower anxiety and depression, together with higher positive self and positive other-beliefs. Similarly, a study found that a PD with Depression group scored significantly higher on negative self-beliefs and significantly lower on positive self-beliefs than the PD group alone^62^. Compared to HCs, PD scored significantly higher on the negative self and negative other-beliefs but did not significantly differ on positive self and positive other-beliefs^50^, indicating that negative self and negative other-beliefs may be more critically related to PD than positive beliefs about self and others. Moreover, childhood emotional abuse was significantly associated with PD, which was mediated by negative other-beliefs^56^, and negative self-beliefs mediated the relationship between sexual abuse and PD in SZ^54^. Additionally, negative other-beliefs significantly and fully mediated the association between loneliness and paranoia, highlighting a relationship between negative core beliefs about others, loneliness and paranoia in SZ^61^.

#### Suicidal risk

Five studies examined the role of core beliefs in suicide risk in SZ^17,24–27^. Except for Self-Sacrifice and Unrelenting Standards, all EMS were significantly positively correlated with suicidal ideation^24^. After controlling for depressive symptoms, the schemas of Emotional Deprivation, Abandonment, Mistrust/Abuse, Social Isolation, Defectiveness, and positive symptoms remained significantly correlated with current suicidal ideation^24^. The Emotional Deprivation schema was correlated with a 1.56 increased risk of lifetime suicide attempts and was significantly associated with positive symptoms, negative symptoms, and depression^24^. Increased negative self and negative other-beliefs^17,25–27^, as well as decreased positive self and positive other-beliefs^25,27^ were significantly correlated with the severity of suicidal ideation in SZ. Childhood trauma and rumination accounted for 49% of the variance in negative schema, and childhood trauma and negative schema accounted for 21.7% of the variance in suicidality^26^. These findings highlight the role of the emotional deprivation schema, as well as increased negative self and negative other-beliefs, along with a decrease in positive self and positive other-beliefs in increasing suicidal ideation and risk in SZ.

#### Recovery

Compared to remitted SZ, recovered SZ demonstrated significantly higher levels of empathy, intact neurocognitive functioning, and positive self-beliefs, highlighting that these aforementioned factors were characteristic of those more likely to achieve recovery^32^.

#### Interventions

Three studies examined interventions targeting core beliefs in SZ^63–65^. A 12-session CBTp intervention was delivered to FEP patients (*n* = 19) and resulted in a significant decrease in positive and negative symptoms, and negative self-beliefs, and a significant increase in positive other-beliefs pre- to post-treatment^63^. A 4-session compassionate imagery intervention was delivered to psychosis patients (*n* = 12) recruited from secondary mental health services (mean age 42 years, SD = 13.1), which resulted in medium effect-size improvements in negative self-beliefs and paranoia, which were maintained at 6 weeks follow-up^64^. Moreover, a 6-session imagery intervention consisting of imagery techniques/imagery rescripting was delivered to FEP patients (*n* = 5)^65^. Clinically significant reductions were found for negative self and negative other-beliefs, persecutory delusions, and imagery distress, suggesting that this imagery intervention was an acceptable and feasible treatment for psychosis^65^. However, it is important to note that this study was uncontrolled and unblinded making it subject to bias, and as there was no follow up it is unclear if the gains were maintained over time. Moreover, as all these interventions were uncontrolled feasibility studies with small sample sizes (ranging between 5–19) lacking in control groups, larger controlled trials are required to examine the effectiveness of these interventions.

#### Qualitative study

Only one qualitative study to date has explored the subjective experience of core beliefs in psychosis, and their relation to hallucinations and delusions^33^. Four emergent themes were identified, namely (1) the solidity and permanency of core beliefs (i.e. that they are long lasting, difficult to change, and drive longstanding behaviours), (2) the development of core beliefs (i.e. negative core beliefs influence psychosis onset, core beliefs are associated with symptoms (hallucinations and delusions), and can influence each other, trauma contributes to negative beliefs, and negative interpersonal experiences shape core beliefs), (3) the synergistic relationship between core beliefs and symptoms (i.e. negative self-beliefs influence symptoms, voices reinforce negative self-beliefs, psychosis can reinforce negative beliefs about others, and positive self-beliefs and psychosis experiences interact in both directions), and (4) core beliefs influence images and psychosis symptoms (i.e. negative beliefs about self/others influence images and voices, negative other-beliefs influence flash-forward persecutory paranoia). These findings elucidate the characteristics of core beliefs in psychosis, which has implications for psychosocial interventions.

### CHR

#### Core beliefs and schemas

Out of the 22 CHR studies included in the present review, only one study employed the YSQ to examine its association with the BCSS negative self/other and positive self/other dimensions^66^. No study to date has examined schema endorsement using the YSQ in CHR compared to healthy controls, nor has the schematic profile between CHR, psychosis, and controls been investigated. All CHR studies in the present review employed the BCSS measure of core beliefs. Compared to HCs, CHR scored significantly higher on negative self^18,53,67,68^ and negative other-beliefs^18,53,67^, and significantly lower on positive self^18,53,67,68^ and positive other-beliefs^18,67^.

#### Trauma

Five studies explored core beliefs and childhood trauma in CHR^18,19,29,30,52^. Compared to HCs, CHR individuals experienced significantly more types of traumas (emotional/physical/sexual abuse, emotional/physical neglect), and bullying^18,29^. Negative self and negative other-beliefs were positively correlated with total trauma and trauma subtypes^29^. Another study found that 72.9% of their CHR sample reported experiencing bullying and had a greater severity of negative other-beliefs, asocial and defeatist performance beliefs, and lower social functioning scores^19^. Negative other-beliefs were significantly higher in the bullied group compared to the non-bullied group^19^. Bullying was significantly positively correlated with negative self^30^ and negative other-beliefs^19,30^. Moreover, negative self and negative other-beliefs were significantly positively correlated with UEDs and significantly mediated the relationship between bullying and UEDs^30^. Additionally, emotional neglect was significantly associated with paranoia and CHR status^18^. Negative self-beliefs mediated both relationships partially^18^. Moreover, after controlling for depression, negative self-beliefs were significantly correlated with physical and psychological abuse, while negative other-beliefs were correlated with psychological bullying and total positive symptoms of psychosis^52^. These findings highlight the high prevalence of trauma and bullying in CHR and the need for interventions to improve maladaptive core beliefs among CHR.

#### Attenuated positive symptoms

Eleven studies examined core beliefs in CHR, and all found significant associations between negative self and negative other-beliefs with attenuated positive symptoms (APS)^30,66,67,69–76^. Compared to HCs, CHR reported significantly more negative self and negative other-beliefs, and significantly less positive self and positive other-beliefs^67^. HCs rarely endorsed negative self-beliefs^67^. These authors also found higher negative self-beliefs and lower positive self-beliefs were associated with depression and APS^67^. Another study examining core beliefs in ARMS and a non-ARMS sample (i.e. individuals presenting with emerging complex mental health difficulties who did not meet the criteria for ARMS) found that the ARMS group scored significantly higher on negative self and negative other-beliefs and significantly lower for positive other-beliefs than the non-ARMS group, with no significant differences found for positive self-beliefs^70^. This non-significant finding regarding positive self-beliefs in the non-ARMS group might be due to the presence of mental health difficulties in this group relative to a HC sample.

In terms of subthreshold positive symptoms in CHR, negative self and negative other-beliefs were associated with APS^66,67,73^, Distressing Unusual Experiences (UED)^30^, suspiciousness^66,67,69–72,74–76^, delusions^71,72^, hallucinations^71^, perceptual abnormalities^66^, unusual thought content^66^, paranormal thinking^71^, and grandiosity^71^.

Core beliefs explained 40–72% of the variance across each UED type (paranoia, hallucinations, delusions, paranormal thinking, and grandiosity)^71^. UED severity was significantly positively correlated with negative self and negative other-beliefs, and negatively correlated with positive other-beliefs^71^. These findings implicate the role of core beliefs across each UED type and in UED severity in CHR.

#### Negative symptoms

Five studies examined core beliefs and negative symptoms in CHR^67,68,77–79^. In CHR, negative self and negative other-beliefs were associated with attenuated psychotic symptoms (particularly negative symptoms and suspiciousness) and depression but not anxiety symptoms^67^. Compared to controls, CHR reported significantly more negative self-beliefs and negative self-esteem, poorer self-concept clarity, and more ruminative self-focus, all of which related to negative symptoms^67,68^. Moreover, negative self-beliefs were associated with asocial beliefs and negative symptoms, while negative self and negative other-beliefs were associated with defeatist performance attitudes^78^. Authors have therefore suggested targeting negative self and negative other-beliefs and attitudes in order to reduce negative symptoms in CHR^78^.

#### Resilience/recovery/transition

One study examined resilience and recovery in CHR^80^. Compared to HCs, CHR demonstrated significantly lower levels of resilience^80^. Higher levels of resilience were associated with reduced negative symptoms, depression and anxiety, and increased levels of role functioning in CHR^80^. Resilient CHR individuals generally reported higher positive self and positive other-beliefs and lower stress to reported life events^80^. One study examined the association between negative self and negative other-beliefs in CHR and the likelihood of transition to psychosis^52^. These authors found that baseline scores for negative self and negative other-beliefs did not significantly differ between those who later transitioned to psychosis compared to those who did not, however those who transitioned had significantly more negative self-beliefs at the time of transition^52^. These collective findings implicate the role of negative self-beliefs in transition to psychosis along with the importance of fostering resilience in CHR to buffer the adverse effects of stress in this group.

### Non-clinical samples

#### Core beliefs and schemas

Out of the 14 non-clinical studies included in the present review, only two employed the YSQ to examine its association with trauma subtypes and psychotic-like experiences^81,82^.

#### Psychotic-like experiences

Nine studies examined core beliefs and PLEs in non-clinical samples and all found significant associations between negative self/other-beliefs on PLEs^31,81,83–89^. Abandonment, Vulnerability, Self-Sacrifice, and Subjugation schemas significantly contributed to voice-hearing^81^. Moreover, negative self and negative other-beliefs significantly and positively predicted Persecutory Ideation (PI)^86,88^, with anxiety partially mediating the relationship between negative self-beliefs and PI but not negative other-beliefs and PI^86^. Additionally, higher negative self-beliefs were associated with a stronger link from paranoia to social anxiety, whilst higher negative other-beliefs were associated with a stronger link from social anxiety to paranoia^84^. These results suggest that negative self and negative other-beliefs appear to modify the relationships between paranoia and social anxiety^84^. Additionally, negative self-beliefs significantly predicted delusion-like experiences, while negative other-beliefs significantly predicted delusion-like experiences and related distress and preoccupation^89^. These authors have therefore suggested that negative other-beliefs may be more critically related to delusion-like experiences than negative self-beliefs in non-clinical samples^89^.

In terms of schizotypy traits, positive schizotypy was significantly associated with negative self and negative other-beliefs, anxiety, depression, and low self-esteem^90^. In contrast, negative schizotypy was associated with diminished positive self and positive other-beliefs^90^. These authors suggest that these findings highlight the differential role of affect in schizotypy, i.e. positive schizotypy characterised by affect dysregulation/high negative affect and negative schizotypy by diminished positive affect^90^.

#### Trauma

Four studies examined core beliefs and trauma in non-clinical samples^81,82,85,91^. Emotional abuse and sexual abuse were significantly associated with the schemas of Defectiveness, Dependency, and Enmeshment^82^. Sexual abuse was significantly and negatively correlated with emotional inhibition, which was significantly and negatively correlated with PLEs^82^. Additionally, sexual and emotional abuse impacted voice-hearing both through the effect of maladaptive schemas (Abandonment, Vulnerability, Self-Sacrifice and Subjugation) and dissociation^81^, while physical abuse impacted voice-hearing only through dissociation^81^. Moreover, PTSD re-experiencing symptoms were most strongly associated with a predisposition to hallucinations, whereas negative self and negative other-beliefs were most strongly associated with a predisposition to paranoia^91^. Negative self and negative other-beliefs were significantly positively correlated with paranoia and hallucinations, while positive self and positive other-beliefs were significantly negatively correlated with paranoia and hallucinations^91^. Lastly, elevated rates of paranoia were only apparent amongst those reporting emotional and physical abuse and were mediated by negative self and negative other-beliefs, as well as depression and anxiety^85^. These findings implicate the role of trauma in the predisposition to psychosis, as well as the role of negative self and negative other-beliefs in the formation of APS in non-clinical samples.

#### Mediators

Four studies examined mediators in the relationship between TLEs and PLEs in non-clinical samples^31,83,85,87^. Greater perceived stress, dissociation, external locus of control, and negative self and negative other-beliefs were significant mediators in the relationship between TLE exposure and higher PLE endorsement^87^. When investigating the independent contribution of each mediator, all five variables significantly mediated the link between TLEs and PLEs^87^. Emotional abuse and physical abuse were associated with elevated rates of paranoia, and these associations were significantly mediated by anxiety, depression, negative self and negative other-beliefs^85^. Moreover, negative self-beliefs and negative other-beliefs, social rank, and loneliness significantly mediated the relationship between social adversity and negative symptoms, whereas, for positive symptoms, only negative self-beliefs and negative other-beliefs were significant mediators^31^. Lastly, in terms of aggression, negative self-beliefs and depression significantly mediated the relationship between indirect aggression and paranoid thinking^83^. In contrast, negative other-beliefs significantly mediated the relationship between direct verbal aggression and paranoid thinking in non-clinical samples^83^. These results emphasise the mediating role of negative self and negative other-beliefs in the association between trauma/social adversity and PLEs in non-clinical samples.

#### Psychological interventions

In a non-clinical sample of university students (*n* = 24) exhibiting high levels of paranoia, a positive/safe interpersonal imagery intervention significantly reduced state and trait paranoia, as well as trait anxiety, and increased positive self-beliefs^92^. Negative/threat imagery significantly increased state paranoia, as well as state and trait anxiety, while neutral imagery reduced positive other-beliefs^92^. These authors suggest that rehearing interpersonal imagery whereby one experiences themselves as safe, secure, and able to trust others may exert positive long-term effects on the individual.

### Meta-analytic findings

#### BCSS: SZ vs. healthy controls

Compared to HCs, meta-analysis of the BCSS in SZ revealed statistically significant large effects for negative self (*d* = 0.91, 95% CI [0.75, 1.07]) and negative other-beliefs (*d* = 0.89, 95% CI [0.59, 1.20]). Statistically non-significant small negative effects were found for positive self (*d* = −0.44, 95% CI [−0.93, 0.05]) and positive other-beliefs (*d* = −0.21, 95% CI [−0.56, 0.13]).

#### BCSS: CHR vs. healthy controls

Compared to HCs, CHR demonstrated statistically significant large effects for negative self (*d* = 0.97, 95% CI [0.29, 1.65]) and statistically significant moderate effects for negative other-beliefs (*d* = 0.74, 95% CI [0.11, 1.37]). A statistically significant moderate negative effect was found for positive self-beliefs (*d* = −0.72, 95% CI [−1.29, −0.16]), while a statistically non-significant moderate negative effect was found for positive other-beliefs (*d* = −0.61, 95% CI [−1.28, 0.07]).

#### YSQ: SZ vs. healthy controls

Compared to HCs, SZ demonstrated statistically significant large effects for twelve out of fifteen schemas. Self-Sacrifice (*d* = 0.50, 95% CI [0.27, 0.72]), and Emotional Inhibition (*d* = 0.76, 95% CI [0.39, 1.14]) demonstrated statistically significant moderate effects, while Unrelenting Standards (*d* = 0.57, 95% CI [−0.01, 1.15]) demonstrated a statistically non-significant moderate effect. The largest effects were found for Mistrust/Abuse (*d* = 1.27, 95% CI [0.58, 1.96]), Social Isolation (*d* = 1.16, 95% CI [0.30, 2.03]), and Emotional Deprivation schemas (*d* = 1.14, 95% CI [0.70, 1.59]. Large effects were found for the following schemas, Vulnerability to Harm (*d* = 0.98, 95% CI [0.39, 1.57]), Entitlement (*d* = 0.98, 95% CI [0.51, 1.45]), Dependence/Incompetence (*d* = 0.97, 95% CI [0.43, 1.52]), Enmeshment (*d* = 0.94, 95% CI [0.71, 1.17]), Defectiveness/Shame (*d* = 0.90, 95% CI [0.51, 1.29]), Failure (*d* = 0.89, 95% CI [0.46, 1.32]), Abandonment (*d* = 0.87, 95% CI [0.57, 1.17]), Subjugation (*d* = 0.86, 95% CI [0.50, 1.23]), and Insufficient Self-Control (*d* = 0.85, 95% CI [0.49, 1.22]).

#### Cognitive model of core beliefs in auditory hallucinations and persecutory delusions

**Fig. 10:**
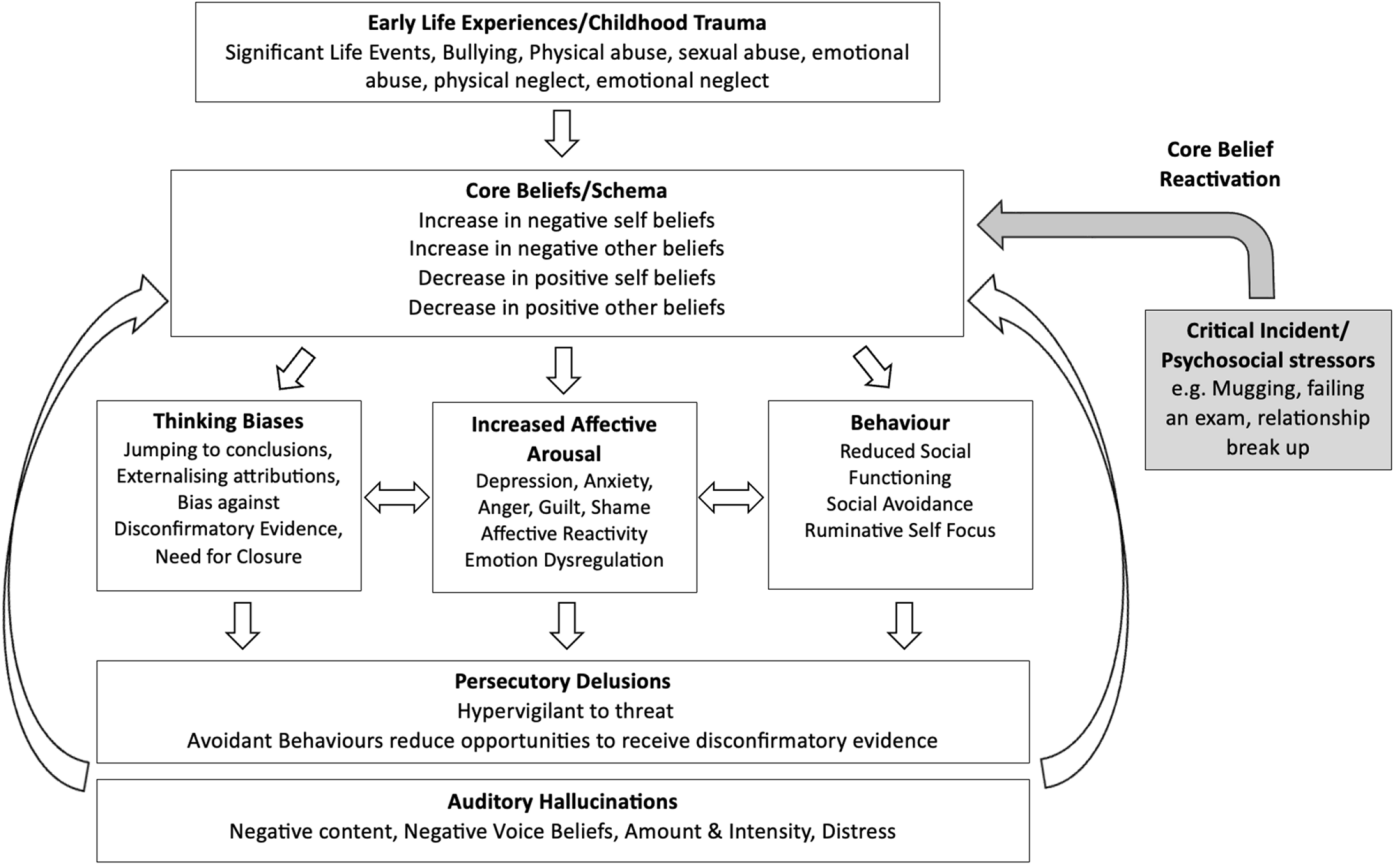
A cognitive model of core beliefs in auditory hallucinations and persecutory delusions.

Based on the findings of the present review, we propose the following *Cognitive Model of Core Beliefs in Auditory Hallucinations and Persecutory Delusions* (see Fig. 10). Our model proposes that early life events/childhood traumatic events such as physical/sexual/emotional abuse and physical/emotional neglect, lead to the development of negative self and negative other-beliefs along with a reduction in positive self and positive other-beliefs. Firstly, core beliefs are likely to influence the role of thinking biases in the processing of information. For example, psychosis specific thinking biases such as jumping to conclusions, an external attributional style, bias against disconfirmatory evidence, and the need for closure will increase the receipt of confirmatory evidence for threatening/distressing beliefs and reject evidence to the contrary, thereby increasing distress. Secondly, the type of core belief about self (e.g. failure, inferior, weak/vulnerable, worthless, unlovable, unlikeable) and others (untrustworthy, uncaring, unreliable, rejecting, dangerous) experienced is posited to induce a high state of negative affect (i.e. sadness, anxiety, anger, guilt, or shame). Thirdly, behaviours such as reduced social functioning, social avoidance, and rumination are likely maintain the conviction of negative beliefs about self and others along with their associated negative affect. Therefore, thinking biases, increased affective arousal, and maladaptive behaviours may influence each other, further magnifying one’s negative emotional state. This subsequently serves as an affective pathway to psychosis via the generation of anomalous experiences. Critical incidents/psychosocial stressors (e.g. mugging, failing an exam, relationship break up) will serve to re-activate pre-existing core beliefs, strengthening the use of maladaptive thinking biases and ensuing distress coupled with behavioural avoidance/rumination, and an increase in negative affect thereby giving rise to the subsequent onset or relapse of psychosis.

Auditory hallucinations may further reinforce the conviction of negative core beliefs about self and others via negative content. Moreover, avoidant behaviours characteristic of those presenting with persecutory delusions will also strengthen pre-existing negative beliefs about self and others due to the lack of opportunities to acquire disconfirmatory evidence to weaken the strength of conviction in those beliefs. Therefore, elucidating the specific core beliefs in psychosis, along with their associated affect, as well as identifying thinking biases, and behavioural coping that cause distress, would offer the opportunity to develop individually tailored cognitive-affective-behavioural-symptom specific formulations in CBT for psychosis to aid in the conceptualisation of psychosis onset and relapse. Therefore, intervention targets for auditory hallucinations and persecutory delusions include addressing negative self and negative other-beliefs, thinking biases, negative affect, social avoidance and rumination, whilst also enhancing positive self and positive other-beliefs, emotional regulation strategies/processing of emotions, and social functioning.

#### Cognitive model of core beliefs in clinical high risk

**Fig. 11:**
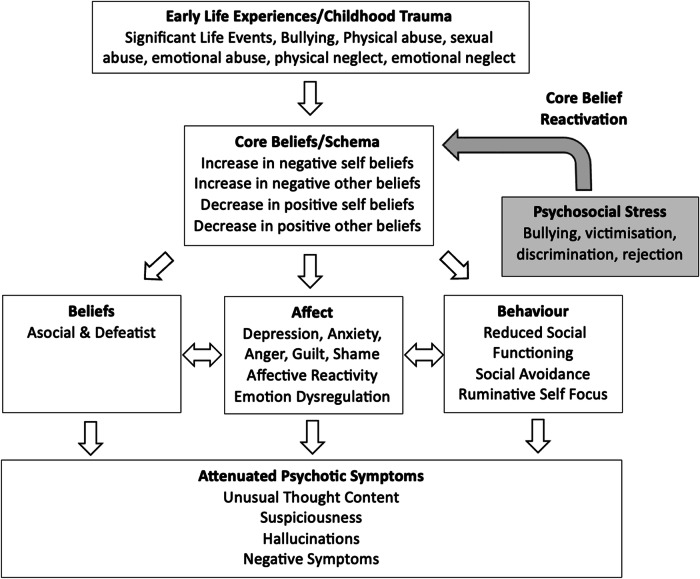
A cognitive model of core beliefs in clinical high risk.

Based on the findings of the present review, we propose the following *Cognitive Model of Core Beliefs in CHR* (see Fig. 11). Our model proposes that childhood traumatic events such as physical/sexual/emotional abuse and physical/emotional neglect, lead to the development of negative self and negative other-beliefs along with a reduction in positive self and positive other-beliefs. The experience of psychosocial stress such as bullying, victimisation, discrimination and rejection serves to reactivate pre-existing core beliefs, which in turn leads to three possible pathways, namely beliefs (asocial and defeatist beliefs), affect (depression, anxiety, worry, affective reactivity, and emotional dysregulation), and behaviour (lowered social functioning, social avoidance, and ruminative self-focus), which all interact to give rise to the generation of subthreshold psychosis symptoms. Therefore, intervention targets for CHR include addressing negative self and negative other-beliefs, asocial and defeatist beliefs, social avoidance and rumination, whilst also enhancing positive self and positive other-beliefs, emotional regulation strategies/processing of emotions, and social functioning.

#### Mediation model for non-clinical samples

**Fig. 12:**
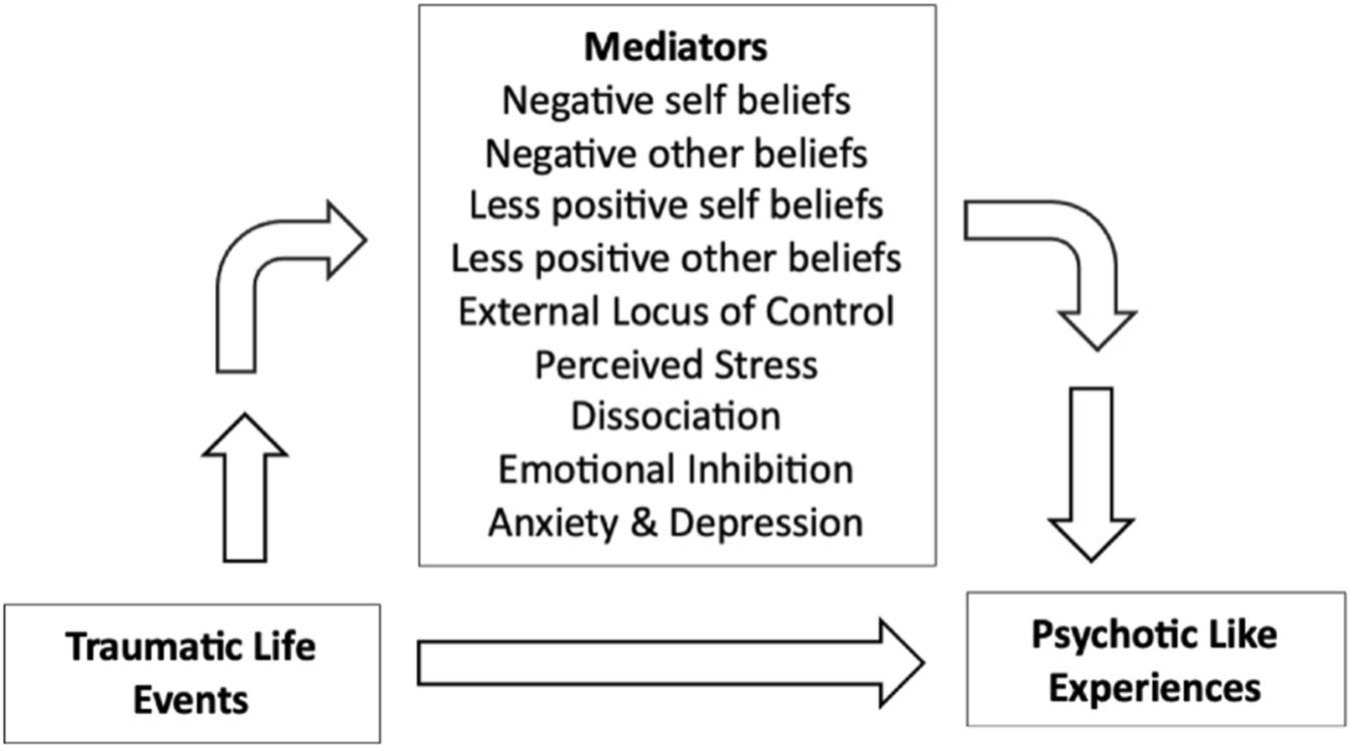
A mediation model for non-clinical samples—mediators for the relationship between TLEs and PLEs.

Based on the findings of the present review, we propose the following *Mediation Model for Non-Clinical Samples* (see Fig. 12). Our model proposes that the relationship between traumatic life events and psychotic-like experiences is mediated by increased negative beliefs about self and others, decreased positive beliefs about self and others, endorsing an external locus of control, higher perceived stress, dissociation, emotional inhibition, as well as increased levels of anxiety and depression. This therefore highlights possible targets for intervention in non-clinical individuals in order to mitigate the emergence of psychotic-like experiences.

## Discussion

This systematic review and meta-analysis sought to investigate the role of core beliefs/schemas in psychosis (SZ and FEP), CHR, and non-clinical samples with PLEs. SZ was characterised by a range of schemas, which were all associated with positive symptoms of psychosis, while only the Social Isolation schema was associated with negative symptoms. These findings highlight the symptom specificity of schemas in psychosis. SZ patients demonstrated a core belief profile characterised by significantly higher negative self and negative other-beliefs and significantly lower on positive self and positive other-beliefs compared to controls. There was also a high prevalence of childhood trauma in the psychosis group, which was also associated with disorganised attachment and positive symptoms of psychosis. These findings highlight the aetiological influence of trauma on insecure attachment styles, maladaptive core beliefs/schemas, and positive symptoms of psychosis. Indeed, trauma/insecure attachment styles, and negative beliefs about self and others have been found across the psychosis continuum. For example, childhood adversity, insecure attachment, and dissociative symptoms correlated with hallucination proneness in a non-clinical sample^93^. Emotional neglect significantly correlated with CHR status and paranoia, both of which were partially mediated by negative self-beliefs^18^. Moreover, childhood emotional trauma was found to significantly predict insecure attachment styles, which in turn predicted negative self-beliefs and subsequent negative AH content in psychosis^14^. These findings implicate the role of core beliefs as a putative mechanism in the relationship between trauma and psychosis symptomatology and highlights the importance of assessing early traumatic events, identifying attachment styles, and elucidating core beliefs/schemas in the development of formulations and effective interventions for distressing AH in psychosis^14^.

According to the cognitive model of positive symptoms of psychosis^12^, childhood trauma creates an enduring cognitive vulnerability, characterised by negative schematic beliefs about the self (e.g. as vulnerable) and the world (e.g. as dangerous) that results in the generation of external attributions and low self-esteem. Birchwood’s cognitive model for auditory hallucinations has also implicated the role of childhood trauma in the formation of negative schemas regarding low social rank and subordination, which in turn influences AH content^94^. Moreover, the cognitive approach to AHs^95^, emphasises the role of beliefs about the voices (i.e. omnipotence, omniscience, and malevolence), rather than the content of the voice in the generation of distress, which is consistent with the findings regarding negative self (e.g. weak, failure) and negative other-beliefs (e.g. dangerous, mistrustful) exhibited in SZ.

Auditory hallucinations in the psychosis group were significantly associated with higher scores for negative self and negative other-beliefs and lower scores for positive other-beliefs compared to controls. Additionally, negative self and negative other-beliefs significantly positively correlated with persecutory beliefs about the voices and the amount and intensity of voice-related distress. Negative self-beliefs significantly contributed to AHs, and the voices in turn, often reinforced negative self-beliefs, indicating a bidirectional relationship between the two. This highlights two possible avenues for intervention, namely addressing the negative self-beliefs, as well as AH content/voice beliefs. For example, addressing negative self-beliefs would lessen the intensity of AHs, and addressing the AH content/voice beliefs would weaken the reinforcement of pre-existing negative self-beliefs.

PDs were characterised by high negative self and negative other-beliefs and low positive self and positive other-beliefs, as well as high levels of depression and anxiety. According to the Cognitive Model of Persecutory Delusions^96^, PDs are conceptualised as threat beliefs occurring in the context of long-term anxiety and depression. A critical incident generates inner-outer confusion and the emergence of anomalous experiences. This in turn leads to one’s search for meaning, whereby pre-existing beliefs about the self and others are drawn upon. These beliefs are based on early life experiences and are typically characterised by beliefs about the self, concerning weakness and vulnerability, and beliefs about others as threatening and hostile. These authors further suggest that negative beliefs about the self, others, and the world contribute to emotional distress, which is then reflected in the content of the delusion. Once formed, the delusion provides confirmatory feedback to affect-related beliefs, further leading to the exacerbation of distress. Indeed, emotional abuse/neglect and poly-victimisation was significantly associated with PDs and delusions of influence, which were all mediated by anxiety^55^. Moreover, negative self-beliefs were found to mediate the relationship between sexual abuse and PD in SZ^54^, while negative other-beliefs significantly and fully mediated the association between loneliness and paranoia^61^, highlighting the mediating role between negative self and negative other-beliefs in the relationship between trauma and PDs.

In contrast to PDs, grandiose delusions were characterised by less negative self-beliefs, and less anxiety and depression, together with higher positive self and positive other-beliefs, and higher self-esteem. These findings reinforce the differences between grandiose and PDs and their emotional states. When controlling for anxiety, physical abuse and poly-victimisation predicted the severity of grandiose delusions highlighting trauma specific associations that were not influenced by anxiety^55^. These findings highlight the differential role of affect in delusional subtypes, which has implications for aetiology and for the development of targeted treatment.

In terms of risk, the Emotional Deprivation schema was correlated with an increased risk of lifetime suicide attempts and was associated with positive symptoms, negative symptoms, and depression. Increased negative self and negative other-beliefs, as well as decreased positive self and positive other-beliefs were significantly correlated with the severity of suicidal ideation in SZ. These findings therefore highlight the importance of addressing maladaptive core beliefs/schemas, not only to alleviate distress associated with symptoms, but to reduce suicidal ideation and attempts in SZ. Moreover, in terms of recovery, recovered SZ demonstrated significantly higher levels of positive self-beliefs compared to remitted SZ, highlighting the important role of positive self-beliefs in achieving remission. Evidence is emerging for the benefits of interventions targeting core beliefs with observations found for a reduction in positive and negative symptoms, and negative self-beliefs, and a significant increase in positive other-beliefs. Although core beliefs have been posited to be enduring cognitive vulnerabilities that are resistant to change, these findings highlight their potential malleability to modification. However, longitudinal studies are required to assess the permanency of core belief changes over time following psychosocial interventions.

The present meta-analysis revealed evidence for the role of negative core beliefs across the psychosis spectrum. Statistically significant large effect sizes were found for negative self (*d* = 0.91) and negative other beliefs (*d* = 0.89) in SZ compared to the statistically significant large and moderate effect sizes observed in the CHR group (*d* = 0.97 and *d* = 0.74, respectively). These findings suggest that negative beliefs about the self and others may be present early in the development of psychosis. The pattern of YSQ findings in SZ are also consistent with the large effects observed in the BCSS. Specifically, Mistrust/Abuse (*d* = 1.27), Social Isolation (*d* = 1.16), and Emotional Deprivation schemas (*d* = 1.14) demonstrated the largest effects, which were all statistically significant. The following schemas demonstrated statistically significant large effect sizes (range *d* = 0.85–0.98; Vulnerability to Harm, Entitlement, Dependence/Incompetence, Enmeshment, Defectiveness/Shame, Failure, Abandonment, Subjugation, and Insufficient Self-Control). Self-Sacrifice and Emotional Inhibition demonstrated statistically significant moderate effect sizes (range *d* = 0.50–0.76), while Unrelenting Standards demonstrated a moderate non-significant effect (*d* = 0.57). These findings point to deeply entrenched interpersonal difficulties in established psychosis. The consistent finding of elevated negative beliefs across both measurement tools (BCSS and YSQ) and clinical populations (SZ and CHR) provides compelling evidence for the centrality of these cognitive structures in psychosis.

The body of studies included were characterised by high heterogeneity, which was identified in the majority of studies, as indicated by high I² values. The YSQ findings extend our understanding by revealing a complex network of maladaptive schemas, with particularly strong effects in domains related to interpersonal trust and social connection, suggesting that these may be key targets for early intervention strategies. The heterogeneity of studies would ideally have been investigated further by meta-regression, however no model had more than the minimum 10 studies recommended by the Cochrane handbook for meta regression, and to perform reliable meta regression analysis^97^.

These meta-analytic findings have significant implications for both theoretical frameworks and clinical interventions. The observation of robust negative belief patterns across both CHR and established psychosis populations supports a vulnerability-stress model where maladaptive core beliefs may serve as early cognitive risk factors rather than merely emerging as a consequence of psychosis. The strong effects observed for interpersonal domains (negative other-beliefs, mistrust/abuse, social isolation) align with contemporary cognitive models of psychosis^34^ and suggest that interventions targeting these specific beliefs/schemas might be particularly beneficial.

A number of clinical methods in CBT have been proposed to address core beliefs such as the two-chair method^98^, imagery techniques^64,65,92^, the continuum technique^99^, addressing cognitive distortions^100^, targeting cognitive, affective, and behavioural responses to schemas^101^, examining the evidence for and against the maladaptive core belief, enhancing more adaptive beliefs, and exploring the advantages and disadvantages of holding maladaptive core beliefs^11^. However, it is unclear which techniques are most effective for psychosis populations and as such future intervention studies examining their feasibility, acceptability, and effectiveness across the psychosis continuum are needed.

Compared to HCs, CHR scored significantly higher on negative self and negative other-beliefs and significantly lower on positive self and positive other-beliefs. CHR also experienced significantly more types of traumas and bullying and were characterised by asocial and defeatist performance beliefs, and lower social functioning. Moreover, negative self and negative other-beliefs significantly mediated the relationship between bullying and UEDs. UED severity was positively correlated with negative self and negative other-beliefs, and negatively correlated with positive other-beliefs. Negative self and negative other-beliefs were also positively correlated with paranoia. These findings might be explained by the *Bio-psychosocial Model of Transition to Psychosis*^34^, which posits that the reactivation of core beliefs in response to psychosocial stress triggers affective arousal and the generation of anomalous experiences in CHR and FEP. Therefore, the experience of bullying may serve to reactivate pre-existing core beliefs concerning threat from others, inducing a high state of negative affect, and the emergence of anomalous experiences. The postulated affective pathway from negative beliefs about self and others to positive symptoms has been supported by a longitudinal structural equation modelling study of non-clinical individuals^102^. These authors found a significant unidirectional longitudinal path from negative beliefs to positive symptoms and a bidirectional longitudinal association from negative beliefs to negative affect and vice versa^102^. These authors concluded that their findings support the postulated affective pathway from negative beliefs to positive symptoms via negative affect, whereby negative beliefs and negative affect influence and amplify each other over several months, leading to the formation of psychosis^102^. These authors, therefore, advocate the importance of interrupting this amplification process by targeting negative beliefs and negative affect early in this trajectory, which could potentially prevent the exacerbation of positive symptoms^102^. Further evidence implicating the role of negative beliefs about self and others in the formation of psychosis pertains to the findings regarding CHR transition to psychosis. For example, although baseline scores for negative self and negative other-beliefs did not significantly differ between those who later transitioned to psychosis compared to those who did not, those who transitioned demonstrated significantly more negative self-beliefs at the time of transition^52^. These findings further implicate the role of negative self-beliefs in the transition to psychosis along with the importance of fostering resilience in CHR to buffer the adverse effects of stress in this group. Moreover, the finding that CHR experienced significantly more types of traumas and bullying compared to HCs also highlights the possible role of trauma interventions for this group. For example, addressing negative beliefs about self and others, as well as trauma appraisals, negative affect, and maladaptive coping may aid in preventing transition to psychosis.

In terms of PLEs in non-clinical samples, schemas concerning Abandonment, Vulnerability, Self-Sacrifice, and Subjugation significantly contributed to voice-hearing while negative self and negative other-beliefs significantly and positively predicted persecutory ideation. Additionally, positive schizotypy was significantly associated with negative self and negative other-beliefs, anxiety, depression, and low self-esteem. In terms of trauma, sexual and emotional abuse impacted voice-hearing both through the effect of maladaptive schemas and dissociation, while physical abuse impacted voice-hearing only through dissociation. These findings highlight the importance of elucidating trauma subtypes, as well as the identification of maladaptive schemas and trauma coping such as dissociation, in order to provide tailored interventions.

Additionally, several factors were found to mediate the relationship between TLEs and PLEs, such as greater perceived stress, dissociation, external locus of control, and negative self and negative other-beliefs, highlighting several modifiable prognostic factors. It has therefore been suggested that targeting stress sensitivity, maladaptive schemas, dissociation, and externalising attributional styles may ameliorate psychosis risk following TLE exposure^87^. It has also been proposed that targeting cognitive vulnerabilities in the form of low perceived social rank, negative self and negative other-beliefs, and loneliness in non-clinical individuals exposed to social adversity could be a promising approach to prevention in non-clinical samples^31^.

### Strengths and limitations

This systematic review and meta-analysis sought to examine the role of core beliefs/schemas in psychosis, CHR, and non-clinical samples with PLEs. Employing a mixed methods synthesis of quantitative and qualitative studies enabled a comprehensive synthesis of the extant findings regarding the role of core beliefs/schemas across the psychosis continuum, which is a strength of the current review^103^. Another strength was the inclusion of studies employing validated measures of core beliefs (BCSS) and schemas (YSQ), which enabled a comparison of findings across studies and clinical populations. From these findings, we have proposed two cognitive models, namely the *Cognitive Model of Core Beliefs in Auditory Hallucinations and Persecutory Delusions* (see Fig. 10), and the *Cognitive Model of Core Beliefs in CHR* (see Fig. 11), all of which can be used to enhance formulation development within CBTp practices, particularly within Early Intervention Services. We have also proposed a mediation model to account for the relationship between TLEs and PLEs in non-clinical samples (see Fig. 12), elucidating potential clinical intervention targets in early psychosis development.

Some limitations of the present study pertain to the risk of not capturing relevant papers from the search outputs despite a manual search also being conducted. Additionally, the current review only included studies published in English and peer-reviewed journals, possibly overlooking applicable research such as grey literature. Twenty-one out of thirty-nine psychosis studies reviewed had sample sizes ranging between 12–82, potentially rendering some studies underpowered to detect statistically significant between-group effects. Core beliefs/schemas were investigated in SZ samples (*n* = 34 studies), CHR (*n* = 22 studies), and in FEP (*n* = 5 studies), yet none of the studies examined a comparison of core beliefs/schemas across these clinical groups, which may reveal as yet unknown core belief/schematic profiles inherent to each group. Also, as most studies (*n* = 73) were cross-sectional, it allowed for correlational analyses to be explored but precluded causal inferences from being made. Moreover, future research would benefit from standardised reporting of effect sizes, public availability of data, and more detailed examination of belief-symptom relationships. Additionally, the meta-analyses included a small subset of studies (range 4–8), which may have rendered some effect sizes statistically non-significant due to insufficient power to detect statistically significant between-group effects. Therefore, more studies with larger samples sizes investigating core beliefs and schemas in CHR and SZ vs. controls are warranted.

### Clinical implications

Core beliefs/schemas were found to play a significant role in the development and maintenance of positive symptoms of psychosis, suicidal ideation and risk, and recovery in individuals with psychosis. The exploration of trauma themes, core beliefs, associated affect, and psychosis symptom-specificity would enhance the development of personalised formulations and targeted interventions in CBTp. Indeed, core belief/schema interventions are demonstrating their emerging promise in reducing positive symptom severity by reducing negative self and negative other-beliefs and by enhancing positive self and positive other-beliefs. The cognitive models proposed within the current review implicate the possible role of core beliefs in auditory hallucinations and persecutory delusions, which have been developed based on the observed pattern of core belief findings in the present review. It is therefore hoped that further research can confirm or refute these theoretical models by employing intervention feasibility studies that explicitly address core beliefs in formulation development and interventions for psychosis within CBTp practices. This would allow for the examination of their effectiveness in reducing psychosis symptoms, negative affect, suicidal risk, and risk of relapse/hospitalisation. Such studies would provide preliminary evidence for their possible clinical utility, which could be subsequently examined in more rigorous randomised control trials with longer term follow ups.

The *Cognitive Model of Core Beliefs in Auditory Hallucinations and Persecutory Delusions* (see Fig. 10) proposes that childhood traumatic events, lead to the development of negative self and negative other-beliefs along with a reduction in positive self and positive other-beliefs. Critical incidents/psychosocial stressors (e.g. mugging, failing an exam, relationship break up) serve to re-activate pre-existing core beliefs, inducing a high state of negative affect and subsequent onset or relapse of psychosis. Testing the clinical utility of this model would be particularly helpful within early intervention services in order to reduce risk of relapse.

The *Cognitive Model of Core Beliefs in CHR* (see Fig. 11) proposes that the experience of psychosocial stress such as bullying, victimisation, discrimination and rejection serves to reactivate pre-existing core beliefs, which in turn leads to three possible pathways, namely beliefs (asocial and defeatist beliefs), affect (depression, anxiety, worry, affective reactivity, and emotional dysregulation), and behaviour (lowered social functioning, social avoidance, and ruminative self-focus), which all interact to give rise to the generation of subthreshold psychosis symptoms. The clinical utility of this model would also benefit from feasibility and randomised control studies examining the effects of explicitly targeting core beliefs in CHR samples and whether it contributes to improvements in mood, functioning, and reduced risk of transition to psychosis. Lastly, the *Mediation Model for Non-Clinical Samples* (see Fig. 12) proposes that the relationship between TLEs and PLEs is mediated by increased negative beliefs about self and others, decreased positive beliefs about self and others, holding an external locus of control, higher perceived stress, dissociation, emotional inhibition, as well as increased levels of anxiety and depression. This therefore highlights possible intervention targets in non-clinical individuals in order to mitigate the emergence of PLEs following TLE exposure, which would also benefit from empirical testing in future research studies. The aforementioned models have important clinical implications as they highlight how identifying and targeting negative self and negative other-beliefs along with their associated affect across the psychosis continuum, would aid in the development of personalised formulations in CBTp. This would not only reduce the severity of positive symptoms of psychosis but would reduce levels of suicidal ideation and risk. Additionally, reducing emotional arousal generated by core belief reactivation would serve to mitigate the affective pathway to psychosis, thereby aiding in relapse prevention and highlighting yet another important intervention target within CBTp. Therefore, facilitating emotional processing, and reducing maladaptive emotion coping whilst also enhancing adaptive emotion coping would be important clinical intervention targets across the psychosis continuum.

### Future directions

Future studies would benefit from the specific examination of negative self-beliefs in psychosis, such as failure, inferiority, weakness/vulnerability, worthlessness, unlovability, and unlikability, along with negative other-beliefs such as mistrust, danger, unpredictability, and unreliability, along with the trauma subtypes that influence their development (physical, emotional, sexual abuse, and physical/emotional neglect). Similarly, the exploration of the type and intensity of emotions generated following core belief reactivation and their associations with specific symptoms of psychosis would be clinically meaningful to further elucidate the affective pathway to psychosis. Future research could helpfully examine core beliefs across CHR, FEP, and more chronic SZ samples, both cross-sectionally and over time to highlight any differences across the trajectory of psychosis, as well identifying their pervasiveness, stability, and association with specific positive and negative symptoms. To date the role of core beliefs and schemas in the development of negative symptoms across CHR, FEP, and SZ is sparse and is thus worthy of further investigation.

There was also only one study to date to examine core beliefs in grandiose delusions, which would benefit from further examination in order to create cognitive models regarding what role core beliefs play in the development of grandiose delusions in psychosis. There is also a notable lack of qualitative studies exploring the role of core beliefs in psychosis. Therefore, elucidating the subjective experience of core beliefs across the psychosis continuum would provide valuable insights into their formation and association with positive and negative symptoms. Additionally, identifying the core beliefs/schemas that are significantly associated with transition to psychosis would enhance CBTp interventions for CHR. Furthermore, the development of psychosocial interventions that explicitly target negative self and negative other-beliefs whilst also enhancing positive self and positive other-beliefs are warranted and would innovate CBTp practices.

## Conclusion

This systematic review and meta-analysis sought to synthesise the existing evidence regarding the role of core beliefs/schemas across the psychosis continuum. Core beliefs/schemas were found to play a significant role in positive symptom, risk, and recovery. They were also found to significantly mediate the relationship between trauma and positive symptoms of psychosis in SZ, and between TLEs and PLEs in non-clinical samples. The *Cognitive Model of Core Beliefs in Auditory Hallucinations and Persecutory Delusions* proposes that childhood traumatic events lead to the development of negative self and negative other-beliefs along with a reduction in positive self and positive other-beliefs. Negative self and negative other-beliefs induce a high state of negative affect, which serves as an affective pathway to psychosis via the generation of anomalous experiences. Therefore, elucidating the specific core beliefs in psychosis, along with their associated affect would offer the opportunity to develop individually tailored cognitive-affective-behavioural-symptom-specific formulations in CBT for psychosis to aid in the conceptualisation of psychosis onset and relapse.

## Supplementary information

Appendix 1

Appendix 2

Appendix 3

Appendix 4

Appendix 5

## Data Availability

All data extracted for meta-analysis and full R code to reproduce our analysis are publicly available here: https://github.com/ricardotwumasi/psychosis-core-beliefs.
