# Supplementary material for: Core beliefs in psychosis: a systematic review and meta-analysis
Source: Schizophrenia (Heidelb). 2025 Mar 6;11(1):38. doi: 10.1038/s41537-025-00577-2 (PMC11885481; doi:10.1038/s41537-025-00577-2)
Supplement: Supplementary file 1 — Appendix 1 [file 41537_2025_577_MOESM1_ESM.docx]

**APPENDIX 1**: **Quality Assessment for Quantitative Studies- EPHPP tool**

| **Author (Year)** | **Selection Bias** | **Study Design** | **Confounders** | **Data Collection Methods** | **Withdrawals and Dropouts** | **Analyses (appropriateness)** | **Global Rating** |
| --- | --- | --- | --- | --- | --- | --- | --- |
| Addington & Tran (2009) | Strong | Strong | Moderate | Strong | Moderate | Yes | Strong |
| Addington et al. (2013) | Strong | Strong | Moderate | Strong | Moderate | Yes | Strong |
| Anilmis et al. (2015) | Strong | Strong | Moderate | Strong | Moderate | Yes | Strong |
| Appiah-Kusi et al. (2017) | Strong | Strong | Strong | Strong | Moderate | Yes | Strong |
| Ashford et al. (2012) | Weak | Strong | Moderate | Strong | Moderate | Yes | Moderate |
| Azadi et al. (2019) | Moderate | Strong | Moderate | Strong | Moderate | Yes | Moderate |
| Barnes et al. (2023) | Moderate | Strong | Moderate | Strong | Moderate | Yes | Moderate |
| Barrantes-Vidal et al. (2013) | Moderate | Strong | Moderate | Strong | Moderate | Yes | Moderate |
| Bennetts et al. (2020) | Moderate | Strong | Moderate | Strong | Moderate | Yes | Moderate |
| Bird et al. (2017) | Moderate | Strong | Moderate | Strong | Moderate | Yes | Moderate |
| Bortolon et al. (2013) | Strong | Strong | Moderate | Strong | Moderate | Yes | Strong |
| Bortolon et al. (2017) | Moderate | Strong | Moderate | Strong | Moderate | Yes | Moderate |
| Boyda et al. (2018) | Moderate | Strong | Moderate | Strong | Moderate | Yes | Moderate |
| Braun et al. (2022) | Strong | Strong | Moderate | Strong | Moderate | Yes | Strong |
| Chau et al. (2023) | Moderate | Strong | Moderate | Strong | Moderate | Yes | Moderate |
| Chung et al. (2021) | Strong | Strong | Moderate | Strong | Moderate | Yes | Strong |
| Chung et al. (2013a) | Moderate | Strong | Moderate | Strong | Moderate | Yes | Moderate |
| Chung et al. (2013b) | Moderate | Strong | Moderate | Strong | Moderate | Yes | Moderate |
| Cole et al. (2017) | Moderate | Strong | Moderate | Strong | Moderate | Yes | Moderate |
| Collett et al. (2016) | Moderate | Strong | Moderate | Strong | Moderate | Yes | Moderate |
| Cowan et al. (2019) | Strong | Strong | Moderate | Strong | Moderate | Yes | Strong |
| Cowan et al. (2024) | Moderate | Strong | Moderate | Strong | Moderate | Yes | Moderate |
| Crowter et al. (2022) | Strong | Strong | Moderate | Strong | Moderate | Yes | Strong |
| Cui et al. (2019) | Strong | Strong | Moderate | Strong | Moderate | Yes | Strong |
| Cui et al. (2020) | Strong | Strong | Moderate | Strong | Moderate | Yes | Strong |
| Davenport et al. (2020) | Strong | Strong | Moderate | Strong | Moderate | Yes | Strong |
| Devoe et al. (2021) | Strong | Strong | Moderate | Strong | Moderate | Yes | Strong |
| Devoe et al. (2022) | Strong | Strong | Moderate | Strong | Moderate | Yes | Strong |
| Fisher et al. (2012) | Moderate | Strong | Moderate | Strong | Moderate | Yes | Moderate |
| Forkert et al. (2022) | Moderate | Weak | Weak | Strong | Strong | Yes | Weak |
| Fowler et al. (2006) | Strong | Strong | Moderate | Strong | Moderate | Yes | Strong |
| Fowler et al. (2012) | Strong | Strong | Moderate | Strong | Moderate | Yes | Strong |
| Freeman et al. (2013) | Strong | Strong | Strong | Strong | Strong | Yes | Strong |
| Freeman et al. (2015) | Strong | Strong | Strong | Strong | Moderate | Yes | Strong |
| Freeman et al. (2019a) | Strong | Strong | Strong | Strong | Moderate | Yes | Strong |
| Freeman et al. (2019b) | Strong | Strong | Strong | Strong | Moderate | Yes | Strong |
| Galbraith et al. (2014) | Moderate | Strong | Strong | Strong | Moderate | Yes | Strong |
| Garety et al. (2013) | Strong | Strong | Moderate | Strong | Moderate | Yes | Strong |
| Gibson et al. (2019) | Moderate | Strong | Moderate | Strong | Moderate | Yes | Moderate |
| Gin et al. (2021) | Strong | Strong | Moderate | Strong | Moderate | Yes | Strong |
| Gracie et al. (2007) | Moderate | Strong | Moderate | Strong | Moderate | Yes | Moderate |
| Hardy et al. (2016) | Strong | Strong | Strong | Strong | Moderate | Yes | Strong |
| Haarmans et al. (2018) | Weak | Strong | Moderate | Strong | Moderate | Yes | Moderate |
| Humphrey et al. (2022) | Moderate | Strong | Strong | Strong | Moderate | Yes | Strong |
| Jaya et al. (2018) | Strong | Strong | Moderate | Strong | Moderate | Yes | Strong |
| Jaya et al. (2017) | Strong | Strong | Moderate | Strong | Moderate | Yes | Strong |
| Khosravani et al. (2021) | Strong | Strong | Strong | Strong | Moderate | Yes | Strong |
| Khosravani et al. (2019) | Strong | Strong | Moderate | Strong | Moderate | Yes | Strong |
| Kusztrits et al. (2022) | Strong | Strong | Moderate | Strong | Moderate | Yes | Strong |
| Lamster et al. (2017) | Moderate | Strong | Moderate | Strong | Moderate | Yes | Moderate |
| LoPilato et al. (2021) | Strong | Strong | Moderate | Strong | Moderate | Yes | Strong |
| MacKinnon et al. (2011) | Moderate | Strong | Moderate | Strong | Moderate | Yes | Moderate |
| Marshall et al. (2016) | Strong | Strong | Moderate | Strong | Moderate | Yes | Strong |
| Marulanda & Addington (2016) | Moderate | Strong | Moderate | Strong | Moderate | Yes | Moderate |
| Michaels et al. (2023) | Strong | Strong | Moderate | Strong | Moderate | Yes | Strong |
| Monsonet et al. (2021) | Strong | Strong | Moderate | Strong | Moderate | Yes | Strong |
| Morrison et al. (2015) | Moderate | Strong | Moderate | Strong | Moderate | Yes | Moderate |
| Müller et al. (2018) | Strong | Strong | Moderate | Strong | Weak | Yes | Moderate |
| Patton et al. (2022) | Moderate | Strong | Moderate | Strong | Moderate | Yes | Moderate |
| Peters et al. (2016) | Moderate | Strong | Strong | Strong | Moderate | Yes | Strong |
| Rammou et al. (2023) | Strong | Strong | Moderate | Strong | Moderate | Yes | Strong |
| Saleem et al. (2014) | Strong | Strong | Moderate | Strong | Moderate | Yes | Strong |
| Scott et al. (2020) | Strong | Strong | Moderate | Strong | Moderate | Yes | Strong |
| Sellers et al. (2018) | Moderate | Strong | Moderate | Strong | Moderate | Yes | Moderate |
| Shahravan et al. (2015) | Weak | Strong | Weak | Strong | Moderate | Yes | Weak |
| Smith et al. (2006) | Strong | Strong | Moderate | Strong | Moderate | Yes | Strong |
| Stowkowy & Addington (2012) | Moderate | Strong | Moderate | Strong | Moderate | Yes | Moderate |
| Stowkowy et al. (2016) | Strong | Strong | Moderate | Strong | Moderate | Yes | Strong |
| Sundag et al. (2016) | Moderate | Strong | Moderate | Strong | Moderate | Yes | Moderate |
| Sundag et al. (2018) | Moderate | Strong | Moderate | Strong | Moderate | Yes | Moderate |
| Taylor et al. (2014) | Strong | Strong | Strong | Strong | Moderate | Yes | Strong |
| Taylor et al. (2017) | Moderate | Strong | Moderate | Strong | Moderate | Yes | Moderate |
| Taylor et al. (2020b) | Weak | Strong | Weak | Strong | Moderate | Yes | Weak |
| Thomas et al. (2015) | Moderate | Strong | Moderate | Strong | Moderate | Yes | Moderate |
| Üçok et al. (2024) | Strong | Strong | Moderate | Strong | Moderate | Yes | Strong |
| Vorontsova et al. (2013) | Strong | Strong | Moderate | Strong | Moderate | Yes | Strong |
| Waite et al. (2019) | Strong | Strong | Moderate | Strong | Moderate | Yes | Strong |
| Zamperoni et al. (2022) | Strong | Strong | Moderate | Strong | Moderate | Yes | Strong |

*Key: EPHPP, Effective Public Health Practice Project*
