# Supplementary material for: Core beliefs in psychosis: a systematic review and meta-analysis
Source: Schizophrenia (Heidelb). 2025 Mar 6;11(1):38. doi: 10.1038/s41537-025-00577-2 (PMC11885481; doi:10.1038/s41537-025-00577-2)
Supplement: Supplementary file 2 — Appendix 2 [file 41537_2025_577_MOESM2_ESM.docx]

**APPENDIX 2: Quality Assessment for Qualitative studies- JBI tool**

| **Author (Year)** | **1. Is there congruity between the stated philosophical perspective and the research methodology?** | **2. Is there congruity between the research methodology and the research question or objectives?** | **3. Is there congruity between the research methodology and the methods used to collect data?** | **4. Is there congruity between the research methodology and the representation and analysis of data?** | **5. Is there congruity between the research methodology and the interpretation of results?** | **8. Are participants, and their voices, adequately represented?** | **9. Is the research ethical according to current criteria or, for recent studies, and is there evidence of ethical approval by an appropriate body?** | **10. Do the conclusions drawn in the research report flow from the analysis, or interpretation, of the data?** | **Include if yes to 2–5, 8–10** |
| --- | --- | --- | --- | --- | --- | --- | --- | --- | --- |
| Taylor et al. (2020a) | Yes | Yes | Yes | Yes | Yes | Yes | Yes | Yes | ✓✓ |

*Key: JBI, Joanna Briggs Institute Quality Assessment Tool (Lockwood et al., 2015).*
