# Supplementary material for: Core beliefs in psychosis: a systematic review and meta-analysis
Source: Schizophrenia (Heidelb). 2025 Mar 6;11(1):38. doi: 10.1038/s41537-025-00577-2 (PMC11885481; doi:10.1038/s41537-025-00577-2)
Supplement: Supplementary file 3 — Appendix 3 [file 41537_2025_577_MOESM3_ESM.docx]

**APPENDIX 3: Characteristics of the Psychosis Sample Studies (n=43)**

| **Author (Year)** | **Country & Type of Study** | **Sample Size & Setting** | **Mean Age (SD)** | **Questionnaires & Diagnostic Tools** | **Main Findings & Clinical Implications** |
| --- | --- | --- | --- | --- | --- |
| Azadi et al. (2019) | Iran Cross-sectional study | 82 SZ (34M/48F) Inpatient | 34.78 (9.10) | The Positive and Negative Syndrome Scale (PANSS)  Young Schema Questionnaire-Short Form (YSQ-SF)  The Beck Depression Inventory-II (BDI-II)  Beck Scale for Suicide Ideation (BSSI) | Positive and negative symptoms, depression, and all EMSs, except for self-sacrifice and unrelenting standards were statistically significantly positively correlated with current suicidal ideation (p<0.001). Emotional deprivation (r=0.32, p<0.01), abandonment (r=0.25, p<0.05), mistrust/abuse (r=0.25, p<0.05), social isolation (r=0.27, p<0.05), defectiveness (r=0.27, p<0.05), and positive symptoms (r=0.29, p<0.05) remained significantly correlated with current suicidal ideation, after controlling for depressive symptoms. Emotional deprivation was correlated with increased odds of lifetimes suicide attempts ((OR) = 1.56, p<0.001). Emotional deprivation was significantly associated with positive symptoms (p<0.01), negative symptoms (p<0.001), and depression (p<0.0001).   Results suggest the potential utility of schema theory (Young 1990) in individuals with schizophrenia who are at risk for suicide. |
| Barnes et al. (2023) | UK  Cross-sectional study | 171 SZ (106M/65F) Outpatient | 42.2 (10.9) | The Trauma History Questionnaire (THQ) The Childhood Experience of Care and Abuse  Questionnaire (CECA-Q)  Hallucination Modalities and Delusion Subtypes.  The Scales for the Assessment of Positive Symptoms (SAPS)  The Brief Core Schema Scale (BCSS)  The Beck Depression Inventory (BDI)39  Beck Anxiety Inventory (BAI) | Significant associations were found between the emotional abuse/neglect and poly-victimization classes with persecutory delusions and delusions of influence, that were all mediated through anxiety (p<0.05). There was an association between the physical abuse class and grandiose/religious delusions that was not explained by the mediators (p< 0.05). Trauma class was not significantly associated with any hallucination modality (p<0.05).  This study demonstrates that childhood victimization is associated with delusions of influence and grandiose beliefs, as well as with persecutory delusions in psychosis. Consistent with previous findings, the potent, mediating role of anxiety supports affective pathway theories and the utility of targeting threat-related processes when treating trauma effects in psychosis. |
| Bortolon et al. (2013) | France  Cross-sectional study | 48 SZ  (32%M/68%F)  44 HC  (28%M/72%F) Outpatient | SZ 37.04 (10.28)  HC 36.95 (13.42) | The Young schema questionnaire short form (YSQ-SF) - the French version of the original The positive and negative syndrome scale (PANSS)  The Beck Depression Inventory-II (BDI-II) | After controlling for depression, patients with schizophrenia achieved higher scores than controls on six EMSs, namely Abandonment, Mistrust/Abuse, Emotional Deprivation, Social Isolation, Defectiveness/Shame, Dependence/Incompetence, Vulnerability to Harm, Enmeshment, Failure, and Subjugation. The EMSs were associated with positive, but not negative, symptomatology. After controlling for depression, only the Mistrust/Abuse schema was a significant predictor of positive symptoms accounting for a small portion (12.4%) of the variance.   The results highlight the importance of focusing not only on the schizophrenic symptoms but also on the person and his or her subjective development of self. Therefore, these results suggest that Young's schema theory may be applied to schizophrenia patients. |
| Chung et al. (2021) | Korea  Cross-sectional study | 500 SZ  (F/M not specified)  (early SZ < 2 years on antipsychotics)  202 HC  (F/M not specified) | Not provided | Brief Core Schema Scale (BCSS) Brooding Scale (BS) Early Trauma Inventory Self Report-Short Form (ETI) Positive and Negative Syndrome Scale (PANSS) Dietary Habits Questionnaire (DHQ) | Compared to HCs, SZ patients scored significantly higher on the following: negative self and negative others on the BCSS, the emotional and cognitive components of the Brooding Scale, general trauma, emotional abuse, physical abuse, and sexual abuse on the ETI, and the DHQ. Emotional abuse was a mediator between trauma and rumination in both groups. However, negative other beliefs mediated the relationship between trauma and rumination in SZ, while negative self-beliefs mediated this relationship for HCs.  The findings suggest that rumination and emotional abuse were central symptoms in both groups and that negative others and negative self-played important mediating roles for SZ and HCs, respectively. |
| Chung et al. (2013a) | Korea  Cross-sectional study | 24 FEP/SZ (19M/8F) Outpatient | 25.67 (4.79) | Positive and Negative Syndrome Scale (PANSS) Psychotic Symptom Rating Scales (PSYRATS) Scale for the Assessment of Negative Symptoms (SANS) Brief Core Schema Scale (BCSS) | This 12 session CBTp intervention resulted in a significant decrease in the BCSS negative-self score (p=0.039) and a significant increase in the BCSS positive others score pre to post treatment (p<0.001). The PSYRATS delusion score was positively correlated with the BCSS negative self (r=0.43, p<0.05) and negative other scores (r=0.58, p<0.01).   Group CBTp for FEP can help decrease negative self-beliefs and increase positive other beliefs. Reducing negative other beliefs and  increasing positive other beliefs may lessen the severity of persecutory delusions in FEP. |
| Chung et al. (2013b) | South Korea Cross-sectional study | 58 SZ Recovered (15M/19F) Remitted (16M/8F) Outpatient | Recovered 33.59 (9.85) Remitted 38.54 (9.26) | Positive and Negative Syndrome Scale (PANSS)  Scale for the Assessment of Negative Symptoms (SANS) Brief Core Schema Scales (BCSS)  Basic Empathy Scale (BES) Schizophrenia Cognition Rating Scale (SCoRS) | Cognitive and total empathy scores, positive self-schemas, and global score on the ScoRS were significantly higher in the recovered than the remitted SZ group. Patients with good levels of empathy and positive self-schema and intact neurocognitive functioning were more likely to achieve recovery.   These results suggest that empathy, positive-self schema and neurocognitive functioning may serve as important clinical characteristics distinguishing those patients who have recovered from those who have achieved only remission. |
| Cole et al. (2017) | UK Cross-sectional study | 180 Voice Hearers  SSD 99 (57%) BD 16 (9%) BPD 13 (7%) Other MH 29 (17%) No MH diagnosis 18 (10%) (68M/111F) Outpatient | 36.65 (11.06) | Hamilton Program for Schizophrenia Voices Questionnaire (HPSVQ) Experiences in Close Relationships – Revised (ECR-R)  Brief Core Schema Scales (BCSS) Beliefs About Voices Questionnaire – Revised (BAVQ-R) | Path analysis provided support for a model in which there were direct pathways from attachment anxiety and avoidance to negative beliefs about self and others; direct pathways from negative beliefs about self and others to persecutory beliefs about voices; and a direct path from persecutory beliefs about voices, and negative beliefs about self, to voice distress. Voice distress positively correlated with negative self-beliefs (r=0.47, p<0.001), negative other beliefs (r=-0.30, p<0.001), and persecutory beliefs (r=0.59, p<0.001). Negative self and negative other beliefs positively correlated with persecutory beliefs (r=0.48, p<0.001 & r=0.44, p<0.001, respectively).  Findings add support to the suggestion that voice-related distress occurs in the context of an insecure attachment style and negative core beliefs about self/others. A therapeutic focus on beliefs about voices, attachment style and core beliefs about self/others may be important to minimize voice-related distress. Further tests of this model that can establish causal relationships between variables are now needed. |
| Collett et al. (2016) | UK Cross-sectional study | 21 PD SZ (10M/11F) 21 HC (10M/11F) Outpatient | PD SZ 45.6 (12.1)  HC 41.9 (12.2) | Psychotic Symptom Rating Scale – Delusions (PSYRATS) Positive and Negative Syndrome Scale (PANSS) Persecution and Deservedness Scale (PaDS) Beck Depression Inventory (BDI) Beck Scale for Suicidal Ideation (BSS) The Self-Compassion Scale (SCS)  The Brief Core Schema Scale (BCSS)  Rosenberg Self-esteem Scale (RSES) | Patients with persecutory delusions had low self- compassion, low self-esteem, increased fears of being mad, beliefs of inferiority to others, negative self-schemas, and low positive self-schemas when compared to the non-clinical control group. SZ PD participants scored statistically significantly lower on positive self-schemas when compared to HCs (p<0.001), and significantly higher on negative self-schemas when compared to HCs (p<0.001). Suicidal ideation was highly associated with negative self-schemas.   This study shows marked negative self-cognitions in patients with persecutory delusions. These are likely to prove targets of clinical interventions, with patient preference most likely determining the best conceptualisation of negative self-cognitions for clinicians to use. |
| Cui et al. (2019) | Korea Cross-sectional study | 314 FEP (135M/179F) | 27.46 (7.21) | Early Trauma Inventory Self Report–Short Form (ETISR-SF) Columbia Suicide Severity Rating Scale (C-SSRS) Calgary Depression Scale for Schizophrenia (CDSS) Positive and Negative Syndrome Scale (PANSS) Brief Core Schema Scales (BCSS) | Independent predictors of recent suicidal ideation included depression, negative schema, and rumination. Furthermore, negative schema and rumination played partial or full mediating roles in the relationship between childhood trauma and recent suicidal ideation.  These findings highlight the importance of performing careful evaluations of childhood trauma and suicidality and of developing effective strategies to reduce mediating factors that may be amenable to psychosocial approaches. |
| Cui et al. (2020) | Korea Cross-sectional study | 306 FEP (145M/161F) | 27.28 (7.11) | Brief Core Schema Scale (BCSS)  Early Trauma Inventory Self Report-Short Form (ETISR-SF)  Columbia Suicide Severity Rating Scale (C-SSRS)  Positive and Negative Syndrome Scale (PANSS) Calgary Depression Scale for Schizophrenia (CDSS) | Childhood trauma accounted for 34.3% of the variance in rumination, and childhood trauma and rumination explained 49% of the variance in negative schema. Childhood trauma and negative schema together accounted for 21.7% of the variance in suicidality. The direct effects of childhood trauma on rumination and negative schema were significant (ß = 0.59, p<0.001 and ß = 0.25, p<0.01, respectively). Also, the direct effects of rumination on negative schema and of negative schema on suicidality were significant (ß = 0.53, p<0.001 and ß = 0.34, p<0.001, respectively).   The findings suggest that targeting negative schema in individuals with FEP exposed to childhood trauma will be an effective strategy for reducing suicidality. |
| Davenport et al. (2020) | UK Cross-sectional study | 44 PD  SZ 16 (36%) SAD 3 (7%) Psychosis 4 (9%) Paranoid SZ 8 (18%) BD 1 (2%) PDs 3 (7%) Multiple diagnoses. 5 (11%) No diagnoses 4 (9%) (22M/22F) Outpatient | 38.9 (14.57) | Structured Interview for Psychosis-risk Syndromes (SIPS) Scale of Psychosis-risk Symptoms (SOPS) Defeatist Performance Attitude Scale (DPAS) Asocial Beliefs Scale (ABS) Social Self-Efficacy subscale from the Revised Self-Efficacy Scale Brief Core Schema Scale (BCSS) | Beliefs about voices correlated with negative voice content and schemas. Negative self-schemas were the strongest predictors of beliefs about voices, whilst positive self-schemas also showed potential relationships. Negative self-schemas correlated with beliefs about voices in terms of malevolence, (0.56, p<0.001), benevolence (-0.42, p<0.01), omnipotence (0.75, p<0.001), metaphysical beliefs (0.61, p<0.001), and loss of control (0.71, p<0.001). Negative other schema correlated with omnipotence (0.47, p<0.001). Positive self-schema correlated with malevolence (-0.31, p<0.05), benevolence (0.30, p<0.05), omnipotence (-0.50, p<0.001), metaphysical beliefs (-0.45, p<0.01), and loss of control (-0.42, p<0.01). Positive other schema correlated with benevolence (0.32, p<0.05) and omnipotence (-0.38, p<0.01).  This study provides evidence that schemas, particularly self-schema, may be important in the development of beliefs about voices. This study offers preliminary findings to suggest that schemas are also associated with the perceived relationship between the hearer and their voice. |
| Forkert et al. (2022) | UK  Feasibility study | 12 SZ  (7M/5F) Outpatient | 42 (13.1) | Green et al. Paranoid Thoughts Scales (GPTS) Self-Compassion Scale Brief Core Schema Scale (BCSS) Social Comparison Scale Rosenberg Self-Esteem Scale | To study examined the feasibility, acceptability, and potential clinical effects of a brief compassionate imagery intervention for patients with persecutory delusions. Post-treatment, there were medium effect size improvements in negative self-schemas and paranoia, Improvements were maintained at 6 weeks follow-up. There were also changes in social comparison, self-esteem, positive self-schemas, and beliefs about others. Post-treatment, there were large effect size improvements in social comparison and self-esteem. There were medium effect size improvements in positive self-beliefs and positive other beliefs. There were large effect size improvements in negative other beliefs, Gains were maintained at 6 months follow-up.   Negative beliefs about the self, including low self-compassion, have been identified as a putative causal factor in the occurrence of paranoia. Therefore, improving self-compassion may be one route to reduce paranoia. |
| Fowler et al. (2006) | UK Cross-sectional study | 252 SZ (72%M/ 38% F) 754 HC (35%M/ 65%F) Outpatient | HC 23.6 (6.5) SZ 38 (10) | Brief Core Schema Scales (BCSS) Rosenberg Self-Esteem Scale (RSES) Young’s Schema Questionnaire – Short Version (YSQ-S) Depression Anxiety Stress Scales (DASS) Paranoia Scale (PS) Peters et al. Delusions Inventory (PDI) | The mean score of 7.2 in the sample of people with psychosis reflects the fact that moderate to strong endorsement of beliefs that ‘I am bad, weak, worthless’ etc. was common. Endorsement of negative beliefs about others (e.g. that others were ‘hostile’, ‘harsh’, ‘unforgiving’, ‘nasty’, etc.) was also common in the psychosis sample. Four variables contributed significantly to the prediction of paranoia in a non-clinical sample. These were, as predicted, in order of importance, negative-other (r=0.57 p<0.0001), positive-other (r=0.49; p<0.0001), anxiety (r=0.48; p<0.0001) and negative-self (r=0.5; p<0.0001). This suggests that the negative-other scale explains approximately 9% of the variance in paranoia, independently of the other variables. Low positive-other scores may account for another 4% and anxiety another 2%, respectively. Positive self-beliefs (p<0.001) and paranoia (p<0.001) contributed to grandiosity beliefs in a non-clinical sample.  Extreme negative evaluations of self and others appear to be characteristic of the appraisals of people with chronic psychosis and are associated with symptoms of grandiosity and paranoia in the non-clinical population. The BCSS may provide a more useful measure of schemata about self and others than traditional measures of self-esteem. |
| Fowler et al. (2012) | UK Cross-sectional study | 301 SZ  (70%M/30%F) | 37.6 (11.0) | Scale for the Assessment of Positive Symptoms (SAPS) Positive and Negative Syndrome Scale (PANSS) Brief Core Schema Scales (BCSS) Beck Depression Inventory-II (BDI-II) | The link between depressed mood and paranoia appeared to be mediated by negative self-schemas. Models based on pathways where the direction was from negative cognition or depressed mood to paranoid symptoms had a better fit than those where the pathways ran in the opposite direction.  This study provides evidence for the role of negative cognition in the maintenance of paranoia, a role of central relevance, both to the design of psychological interventions and to the conceptualizations of psychosis. |
| Freeman et al. (2013) | UK Cross-sectional study | 130 SZ (82M/48F) Outpatient | 41.1 (11.6) | Brief Core Schema  Scale (BCSS) Scales for the Assessment of Positive Symptoms (SAPS) Beck Anxiety Inventory (BAI) Beck Depression Inventory-II (BDI-II) Threat Anticipation Interpretation of Ambiguity | Higher levels of state paranoia were associated with higher ratings of anticipation of threat to the self, negative interpretations of ambiguous events, private and public self-consciousness, and negative self-beliefs. Paranoia was unrelated to anticipating positive or neutral events occurring to the self or to any type of event happening to another person, i.e., there was a specific association of paranoia with anticipating negative events occurring to the self.   Reducing negative ideas about the self and encouraging more positive views will lead to improvements in levels of paranoia. |
| Freeman et al. (2015) | UK RCT | 59 PD total  28 Street Exposure (14M/14F)  31 Neutral Task condition (21M/10F) | Street Exposure 43.8 (10)  Neutral Task 42.9 (11.5) | Brief Core Schema Scales (BCSS) Schizotypal Symptoms Inventory—Paranoia (SSI) Maudsley Assessment of Delusions Scale (MADS) Scale for the Assessment of Positive Symptoms (SAPS) Scale for the Assessment of Negative Symptoms (SANS) | This study compared the effects on patients of going outside into a busy social environment with staying inside. Compared with staying inside, the street exposure condition resulted in significant increases in paranoia, voices, anxiety, negative self-beliefs and negative other beliefs. There was also a decrease in positive self-beliefs. There were indications that the increase in paranoia was partially mediated by increases in anxiety (45%), depression (38%), and negative beliefs about others (45%).  Increases in negative affect may form an important route by which social exposure in urban environments triggers paranoid thoughts. The study provides an illustration of how an experimental approach can be applied to help understand a specific difficulty for patients with psychosis. In future studies the effects of specific elements of the social environment could be tested. |
| Freeman et al. (2019a) | UK Cross-sectional study | 1809 SZ (1255M/547F) Outpatient | 41.3 (12.9) | Green et al Paranoid Thoughts Scale (GPTS) Cardiff Anomalous Perceptions Scale-hallucinations (CAPS) Penn State Worry Questionnaire (PSWQ) Brief Core Schema Scales (BCSS) | Paranoia was significantly correlated with higher levels of anxious avoidance (r=0.37, p<0.001), worry (r=0.40, p<0.001), negative self-beliefs (r=0.47, p<0.001), insomnia (r=0.41, p<0.001), and hallucinations (r=0.54, p<0.001) and lower levels of positive self-beliefs (r=−0.17, p<0.001), rational reasoning (r=-0.14, p<0.001), and psychological wellbeing (r=-0.29, p<0.001). Hallucinations were significantly correlated with higher levels of anxious avoidance (r= 0.41, p<0.001), worry (r=0.39, p<0.001), negative self-beliefs (r=0.42, p<0.001), insomnia (r=0.39, p<0.001), and paranoia (r=0.54, p<0.001), and with lower levels of positive self-beliefs (r=−0.20, p<0.001), rational reasoning (r=-0.23, p<0.001) and psychological wellbeing (r=−0.30, p<0.001).   The study highlights the need for interventions targeting anxious avoidance, worry, low self-esteem, negative self-core beliefs, and insomnia and other transdiagnostic mechanisms to improve psychosis symptoms such as paranoia and hallucinations. |
| Freeman et al. (2019b) | UK Cross-sectional study | 110 SZ (65M/45F) Outpatient | 42.3 (11.5) | Columbia-Suicide Severity Rating Scale (C-SSRS) Beck Depression Inventory-II (BDI-II) Psychotic Symptom Rating Scales – Delusions (PSYRATS) Persecutory Delusions (SBQ) Warwick-Edinburgh Mental Well-being Scale (WEMWBS) | The psychological processes linked to severity of suicidal ideation were negative beliefs about the self and others, fewer positive beliefs about the self and others, pessimism, worry, lower anticipation of pleasure, and paranoia-related defence behaviours.  Patients with persecutory delusions are typically in a severe state of psychological stress, and at risk of suicide, as indicated by very high levels of suicidal ideation. |
| Garety et al. (2013) | UK  Cross-sectional study | 134 Persecutor only (91M/43F) 39 Grandiose only (31M/8F) 58 Both Persecutory & Grandiose (48M/10F) Neither Persecutory or Grandiose (31M/18F) Outpatient | Persecutory only 39.5 (11.0)  Grandiose only 33.0 (9.3) Both Persecutory & Grandiose 37.1 (10.0) Neither Persecutory or Grandiose 35.3 (8.9) | The Brief Core Schema Scale (BCSS)  Positive and Negative Syndrome Scale (PANSS) Scale for the Assessment of Positive Symptoms (SAPS) Beck Depression Inventory (BDI-II) Rosenberg Self-Esteem Scale (RSES) | The Persecutory Only group had higher negative self and negative other schemas, and lower positive self and positive other schemas compared to the other clinical groups. The Grandiose Only group had the highest positive self and positive other schemas and the lowest negative self-schemas. Negative self-evaluations and depression and anxiety predicted a significantly increased chance of persecutory delusions whereas grandiose delusions were predicted by less negative self-evaluations and lower anxiety and depression, along with higher positive self and positive other evaluations.  The significant differences in the processes associated with these two delusion subtypes have implications for aetiology and for the development of targeted treatment strategies. |
| Hardy et al. (2016) | UK Cross-sectional study  Baseline, 3, 6, & 12 month follow up | 228 SZ (165M/63F) Inpatient & Outpatient | 38.24 (11.11) | Trauma History Questionnaire (THQ)  Scales for the Assessment of Positive Symptoms (SAPS)  Self-report Scale for Posttraumatic Stress Disorder (SRS-PTSD) Brief Core Schema Scale (BCSS) Beck Depression Inventory II (BDI-II). | Key findings indicated that 74.1% of participants reported victimisation trauma, and 21.5% met symptomatic criteria for PTSD. There was a specific link between childhood sexual abuse and auditory hallucinations (OR = 2.21, p=0.018), mediated by post traumatic avoidance and numbing (OR =1.48, p=0.038) and hyperarousal (OR =1.44, p=0.045), but not intrusive trauma memory, negative beliefs or depression. Childhood emotional abuse was statistically significantly associated with persecutory delusions (OR = 2.21, p=0.009) and referential delusions (OR = 2.43, p=0.004), with the former mediated by negative other beliefs (OR = 1.36, p=0.024) but not posttraumatic stress symptoms, negative-self beliefs, or depression. There was no evidence of mediation for referential delusions. No relationships were identified between childhood physical abuse and psychosis.  The findings underline the role of cognitive-affective processes in the relationship between trauma and symptoms, and the importance of assessing and treating victimization and its psychological consequences in people with psychosis. |
| Haarmans et al. (2018) | Canada  Cross-sectional study | 44 SZ AVH (44F) 48 HC  (48F) | SZ AVH 42.14 (13.38) HC 33.90  (12.83) | Structured Clinical Interview for DSM-IV Disorders (SCID) Positive and Negative Symptom Scale (PANSS) Childhood Trauma Questionnaire (CTQ) Gender Role Stress Scale (GRS) Brief Core Schema Scales (BCSS) | Compared to HCs, the SZ AVH group had higher scores for negative self-schemas (p<0.02), and negative other schemas (p<0.005), and lower scores for positive other schemas (p<0.02).  In general, study findings implicate the importance of attending to the impact of gender and culture on schematic beliefs in order to enhance case formulations and psychological interventions for women with psychosis. |
| Humphrey et al. (2022) | UK Cross-sectional study | 242 SZ (154M/74F/ 14 Other) Outpatient | 33.17 (13.06) | Brief Betrayal Trauma Survey (BBTS) Psychosis Attachment Measure – Revised (PAM-R) Brief Core Schema Scales (BCSS) Persecutory and Deservedness Scale (PaDS) Community Assessment of Psychic Experiences (CAPE) | The study highlighted a high prevalence of childhood interpersonal trauma, with 89% reporting at least one trauma. Childhood interpersonal trauma was associated with disorganised attachment (p=0.002), which in turn was associated with negative self-schema (p<0.001), negative other schema (p=0.009), and paranoia (p=0.001). Disorganised attachment and negative other beliefs fully mediated the relationship between trauma and paranoia. Negative other beliefs partially mediated the association between disorganised attachment and paranoia. Results were found when controlling for depression, hallucinations, and age.  Results suggest that interventions that aim to modify disorganised attachment patterns and negative beliefs about others can potentially alleviate the impact of trauma on paranoia. Findings provide justification for longitudinal studies to confirm the direction of effects, and intervention studies that aim to manipulate disorganised attachment and negative schema about others and observe the impact of this on paranoia. |
| Jaya et al. (2017) | Germany, Indonesia,  USA  Longitudinal cohort study | 962 SZ (54.4M/45.6F) Outpatient | 34.4 (11.85) | Brief Core Schema Scale (BCSS) Generalized Anxiety Disorder-7 scale (GAD-7) Negative Affect: Defined as the combined sum-score of the PHQ-9 and GAD-7 Community Assessment of Psychic Experiences (CAPE) | These authors found a significant unidirectional longitudinal path from Negative Self Schemas (NSS) to positive symptoms (p<0.01) and bidirectional longitudinal associations from NSS to negative affect (p<0.01) and vice versa (p<0.01). There was also a significant indirect pathway from NSS at baseline via negative affect at T1 and T2 to positive symptoms at T3, indicating mediation.  The findings support the postulated affective pathway from negative self-schemas to positive symptoms via negative affect. Specifically, the data indicated that negative self-schemas and negative affect influence each other and build up over the course of several months before leading on to positive symptoms. They conclude that interrupting this process by targeting negative self-schemas and negative affect early in the process could be a promising strategy to prevent the exacerbation of positive symptoms. |
| Khosravani et al. (2019) | Iran  Cross-sectional study | 105 SZ (43M/62F)  90 non-patients with high schizotypal traits (30M/60F) 90 non-patients with low schizotypal traits (36M/54F) Inpatient & outpatient | SZ: 32.99 (9.99) non-patients with high schizotypal traits: 18.67 (1.40) Non-patients with low schizotypal traits: 18.97 (2.51) | Young Schema Questionnaire-Short Form (YSQ-SF) Positive and Negative Syndrome Scale (PANSS) Schizotypal Personality Scale (STA) Beck Depression Inventory-II (BDI-II) | Mistrust/abuse and social isolation schemas were significant predictors of positive and negative symptoms in SZ patients respectively. SZ patients and non-patients with high schizotypal traits exceeded non-patients with low schizotypal traits on all EMSs. There were no differences between SZ patients and non-patients with high schizotypal traits regarding EMSs. In the subgroups with high depression, SZ patients showed higher levels of EMSs than non-patients with low and high schizotypal traits. In the subgroups with low depression, both SZ patients and non-patients with high schizotypal traits had higher scores on EMSs than non-patients with low schizotypal traits.  The findings suggest that mistrust/abuse and social isolation may be specific to psychosis symptoms. Also, SZ and schizotypal traits may overlap in relation to EMSs. In addition, the activated EMSs may contribute to high depression in SZ. |
| Khosravani et al. (2021) | Iran Cross-sectional study | 82 SZ (34M/48F)  120 OCD (58M/62F) 100 BD (57M/43F)  60 HC (30M/30F) Outpatient | OCD 33.87 (12.64) BD 36.66 (8.51) SZ 34.78 (9.10) HC 28.57 (11.14) | Young Schema Questionnaire-Short Form (YSQ-SF) Beck Depression Inventory-II (BDI-II) Yale-Brown Obsessive Compulsive Scale (Y-BOCS) Young Mania Rating Scale (YMRS) Positive and Negative Syndrome Scale (PANSS) | Schizophrenia patients scored higher on Mistrust/Abuse (p<0.001) than OCD and BD patients. Patients with OCD, BD, and schizophrenia had higher scores on all schemas compared to HCs. OCD patients scored higher on all schemas except entitlement, insufficient Self-control, and mistrust/abuse compared to individuals with schizophrenia and/or BD. The entitlement and insufficient self-control schemas were higher in BD patients than in patients with OCD and schizophrenia. Similar findings were also reported in subgroups with low and high depression.  The findings show that some of the schemas are specific to OCD, BD, and schizophrenia. Also, patients with OCD, BD, and schizophrenia have the highest numbers of schemas respectively. In addition, increased depression may activate EMSs in patients with OCD, BD, and schizophrenia. |
| Kusztrits et al. (2022) | Australia Cross-sectional study | 76 Non-affective VH (30M/46F) 65 SZ VH (30M/35F) 33 Non-clinical VH (15M/18F) Inpatient & Outpatient | 35.27 (12.63) - Total | Mini International Neuropsychiatric Interview Screen (MINI) Launay–Slade Hallucinations Scale Wechsler Test of Adult Reading (WTAR) Beck Depression Inventory II (BDI-II) Beck Anxiety Inventory (BAI) Brief Core Schema Scale (BCSS) Questionnaire for Psychotic Experiences (QPE) | Negative core schemas are positively associated with the amount and intensity of voice-related distress due to AVHs. Affective and non-affective voice hearers differ in terms of voice frequency and the impact on functioning, but not in terms of depression, anxiety, AVH distress and core schemas about the self and others. The core schemas–AVH distress relationship is mediated by depression in non-affective voice hearers.  These findings suggest that schemas influence emotional aspects of AVH phenomenology differently depending on the underlying psychopathology. Future integrative approaches will be useful to uncover the complex influence of emotions and their related processes to reveal potential targets for the treatment of transdiagnostic AVHs arising from different underlying psychopathologies. |
| Lamster et al. (2017) | Germany, Switzerland Cross-sectional study | 65 SZ  (43.1% M/56.9%F) Outpatient | 40.49 (12.6) | Brief Core Schemas Schema (BCSS) University of California Loneliness Scale (UCLA-LS) Paranoia Checklist (PCL-FR, PCL-DIS) Frequency of Social Contact: Frequency of Social Contact Scale (FOSC) | The association between loneliness and paranoia was significantly and fully mediated by negative schemas of others. Moreover, a low level of perceived social support was significantly associated with loneliness, whereas self-reported frequency of social contact was not.   The present results highlight the potential role of interpersonal negative schemata in the formation and maintenance of paranoia and elucidate the crucial role of loneliness in the way individuals construe themselves within a social environment. |
| MacKinnon et al. (2011) | UK Cross-sectional study | 16 PLES (14F/2M) 20 HC (8M/12F) Outpatient | PD 41.69 (11.09) 20 HC 29.50 (11.42) | Structured Clinical Interview for DSM-IV-TR Axis I disorders (SCID-I) Implicit Self-Esteem- Implicit Association Test (IAT) Explicit Self-Esteem: Rosenberg Self-Esteem Scale (RSE) Positive and Negative Self and Other Beliefs: Brief Core Schema Scales (BCSS) | There was no significant difference between the groups on the measure of positive-self or positive-other. The persecutory delusion group scored significantly higher on the negative-self subscale and the negative-other subscale (p<0.01) than the control group. This indicates that the persecutory delusion group held more negative views of themselves and of others than the control group. The persecutory delusion group scored significantly lower than the control group on the RSE (p<0.01), indicating lower explicit self-esteem. However, DASS-21 scores as a covariate eliminated the difference in explicit self-esteem scores between the groups (p=0.65). This suggests that the variation between the persecutory delusion and control group on explicit self-esteem may be partly accounted for by levels of social anxiety, depression and anxiety.  The results do not support the contention that persecutory delusions defend against negative self-representations and low self-esteem reaching conscious awareness. Non-defensive cognitive models are discussed as an alternative way of understanding persecutory delusions. |
| Peters et al. (2016) | UK Cross-sectional study | 84 Psychotic Disorders with Persistent Psychotic Experiences (PE) (55M/29F) 92 Non-Clinical Group (never diagnosed but with PE) 83 Non Clinical Controls with no PE (26M/57F) Inpatient & outpatient | PE Non-clinical group: 46 PE Clinical group: 42  Control group: 46 (SD not provided) | Brief Core Schemas Scale (BCSS) Structured Interview for Prodromal Symptoms (SIPS) Global Functioning Scale (GFS) Beck Depression Inventory-II (BDI-II) Psychosis Screening Questionnaire (PSQ) Unusual Experiences Screening Questionnaire (UESQ) Scale for the Assessment of Positive Symptoms (SAPS) Scale for the Assessment of Negative Symptoms (SANS) Victimization Experiences Schedule (VES) | Results showed that the clinical group scored lower than the non-clinical and control groups on positive self-schemas (p<0.001). For negative self and negative other schemas, the clinical group scored higher than the non-clinical and control groups (p<0.001 for both). For positive other schemas, the clinical group scored lower than the non-clinical and control groups (p=0.006). Overall, these findings suggest that individuals in the clinical group had more negative and fewer positive core self and other schemas when compared to those in the Control and Non-clinical groups.  These findings have potential implications for the clinical management of people with PEs, including individuals at UHR for psychosis. Psychological therapies (including CBT for psychosis and third-wave therapies) all have a normalizing and accepting approach to PEs as a central tenet. Since PEs can occur without pathological outcomes, the aim of therapy may not necessarily be to eliminate such experiences, but to appraise them in a less threatening and paranoid way, or to deal with them differently. |
| Rammou et al. (2023) | UK Cross-sectional study | 34 FEP (7M/25F/2 Other) 34 HC (9M/25F) |  | Comprehensive Assessment of At-Risk Mental States (CAARMS) Structured Clinical Interview for DSM-IV Axis I Disorders (SCID-I-RV)  Mini International Neuropsychiatric Interview (MINI)  Persons Relating to Others Questionnaire (PROQ-3)  Brief Core Schema Scales (BCSS) | Voice-hearers scored higher on negative self (p=0.001) and negative other schemas (p=0.002), and depressive and anxiety symptoms (p<0.001). The two groups did not differ significantly on childhood trauma levels, however, the voice-hearing group scored lower on premorbid adjustment (p<0.001).  Hearing voices in help-seeking youth could be an indicator for social relating issues and holding negative schematic beliefs and may be an indicator for of increased psychopathological complexity. Although poorer premorbid adjustment might indicate an early vulnerability to social relating difficulties, voice-hearing might be an aggravating factor and one that requires treatment. |
| Scott et al. (2020) | Australia  Cross-sectional study | 140 Patients with AVH (SZ, SZA, MDD, BD) (60M/80F) Inpatient & outpatient | 36.7 (12.83) | Mini International Neuropsychiatric Interview Screen (MINI) Childhood Trauma Questionnaire (CTQ) Psychosis Attachment Measure (PAM) Brief Core Schema Scale (BCSS) | Negative AVH content was significantly predicted by negative self-schemas. In turn, negative self-schemas were significantly predicted by insecure anxious attachment and childhood emotional neglect. Anxious attachment fully mediated the relationship between childhood emotional abuse and negative self-schema. Emotional abuse and neglect were the only childhood trauma dimensions that contributed significantly to the model. Collectively, emotional abuse and neglect, insecure anxious and avoidant attachment, and negative self-schemas accounted for 10% of the variance in the proportion of negative AVH content experienced by voice hearers.  This study marks an important step towards understanding the underlying mechanisms involved in negative AVHs. The results highlight the importance of recognising early experiences of trauma, adult attachment styles and self-schemas in developing formulations and effective treatments for negative and distressing AVHs. Psychological interventions that target these underlying mechanisms of negative AVHs may lead to a reduction in negative content, thereby reducing voice related distress. |
| Shahravan et al. (2015) | Iran  Cross-sectional study | 20 SZ 20 BD 20 drug-induced psychosis (60M) Inpatient | Not specified | Early Maladaptive Schemas (YSQ_SF) Peters Delusional Inventory (PDI) | The SZ group consistently scored highest in most schemas, including Emotional Deprivation, Abandonment, Social Isolation, Defectiveness/Shame, Dependence, Vulnerability, Enmeshment, Subjugation, Emotional Inhibition, Insufficient Self-Control, Continence, and Total Schema score. The study found statistically significant correlations between the mistrust/abuse schema and delusional beliefs (discomfort, confidence) in SZ. BD showed significant correlations between delusional beliefs in three dimensions with the mistrust/abuse schema. Substance-induced psychosis patients exhibited significant correlations between delusional beliefs in three dimensions with Mistrust/Abuse, Failure, and Vulnerability. The schema of Mistrust/Abuse had a positive and significant relationship with delusional beliefs in all three groups. Individuals with Mistrust/Abuse schema may have experienced physical and sexual abuse, repeated punishment, and unsafe living environments.  In general, current result show that early maladaptive schemas have significant correlation with delusional beliefs in psychotic patients. |
| Smith et al. (2006) | UK  Cross-sectional study | 100 SZ (68M/32F) Outpatient | 39 (10.9) | Scale for the Assessment of Positive Symptoms (SAPS) Positive and Negative Syndrome Scale (PANSS) Psychotic Symptom Rating Scales (PSYRATS) Beck Depression Inventory-II (BDI-II) Rosenberg Self-esteem Scale (RSES) Brief Core Schema Scales (BCSS) | Analysis indicated that individuals with more depression and lower self-esteem had auditory hallucinations of greater severity and more intensely negative content and were more distressed by them. In addition, individuals with more depression, lower self-esteem and more negative evaluations about themselves and others had persecutory delusions of greater severity and were more preoccupied and distressed by them. The severity of grandiose delusions was related inversely to depression scores and negative evaluations about self, and directly to higher self-esteem.  This study provides evidence for the role of emotion in schizophrenia spectrum-disorders. Mood, self-esteem and negative evaluative beliefs should be considered when conceptualising psychosis and designing interventions. |
| Sundag et al. (2016) | Germany  Cross-sectional study | 81 SZ  (54%M/46%F)  28 DEP (46%M/54%F) 60 HC (33%M/67%F) Inpatient & outpatient | SZ 36.4 (11.8)  DEP 36.7 (10.2)  HC 36.9 (13.6) | Mini International Neuropsychiatric Interview (MINI)  Positive and Negative Syndrome Scale (PANSS)  Young Schema Questionnaire-Short Form (YSQ-SF) | Compared to the HCs, patients with psychosis or depression showed a higher overall number and intensity of EMS whereas the psychosis and the depression sample did not significantly differ from each other. The overall number and intensity of EMS were significantly associated with positive but not with negative symptoms. Contrary to previous findings, patients with psychosis and patients with depression did not differ in the EMS subscale Mistrust/Abuse. The results suggest that EMS are particularly relevant to positive symptoms.  These findings imply that addressing maladaptive schemas in patients with psychosis by making use of the schema-concept holds potential. |
| Sundag et al. (2018) | Germany Cross-sectional study | 20 PD (40%M/60%F) 40 HC (32.5%M/ 67.5%F) | PD 38.70 (12.87)  HC 40.03 (10.78) | Mini International Neuropsychiatric Interview (MINI) Positive and Negative Syndrome Scale (PANSS)  Young Schema Questionnaire-Short Form (YSQ-SF)  Cyberball paradigm (social stress induction) | Patients with PD responded with a stronger increase in paranoia and revealed a significantly higher EMS total score compared to the healthy controls. Higher increases in paranoia following the social stress were accounted for by higher EMS total scores. Specific schemas, such as Defectiveness/Shame (r(57)=0.456, p<0.001) and Enmeshment/Undeveloped Self (r(57)=0.436, p<0.001), were associated with increases in paranoia following social stress.  EMSs are associated with stress‐related symptom increases in patients with PD.  The findings suggest that addressing EMSs in psychological treatment of patients with PD holds potential. |
| Taylor et al. (2014) | UK  Cross-sectional study | 20 FEP (73.7%M/ 26.3%F) 113 ARMS (59.3%M/ 40.7%F) 28 Help-Seeking Clinical Group (HSC) (82.1%M/ 17.9%F)  30 NH (Non-Help-Seeking Individuals with PLES) (26.7%M/ 73.3%F) Outpatient | FEP 22.4 (5.4)  ARMS 20.4 (4.3) HSC 21.3 (3.4)  NH + PLES 22.8 (3.7) | The Comprehensive Assessment for At Risk Mental States (CAARMS) Brief Core Schema Scales (BCSS) | The clinical groups scored significantly higher than the NH group for negative beliefs about self and about others. No significant effects of group on positive beliefs about others were found. For positive beliefs about the self, the NH group scored significantly higher than the clinical groups. Furthermore, negative beliefs about self and others were related to positive psychotic symptomatology and to distress related to those experiences.  The findings suggest that elevated levels of negative beliefs about the self and others are prominent in the FEP and ARMS populations and are associated with a range of positive psychotic experiences and the distress that results from those experiences. As psychological interventions are seen as more ethical over medication for the ARMS population, core schemas are likely to be an important target for such interventions. |
| Taylor et al. (2017) | UK  Cross-sectional study | 20 SZ (13M/7F) Outpatient | 44 (11.4) | Young’s Schema Questionnaire—Short Version (YSQ-S)  Social Functioning Scale (SFS)  Clinical Outcomes In Routine Evaluation (CORE) | Dependency and enmeshment schema were significantly inversely associated with social functioning with a moderate effect size for both (rs = −0.50; −0.54, respectively). Eight schemas were significantly associated with distress. Significant correlations were found between CORE distress and the following EMSs: (Abandonment, Mistrust/Abuse, Social Alienation, Failure, Dependency, Vulnerability to Harm, Enmeshment, and Subjugation of needs).    These results suggest that early maladaptive schema may have an important role in psychosis and could be considered as part of psychological therapies that seek to enhance social functioning and reduce distress. |
| Taylor et al. (2020a) | UK  Qualitative study | 20 SZ (15M/5F) Outpatient | 31.49 (9.0) | PANSS Delusions or Hallucinations subscales Thematic analysis | Four emergent themes were identified: (i) the solidity and permanency of core beliefs indicating that these beliefs are resistant to change, enduring, and influence long-term behaviours, (ii) the causes and development of core beliefs show that negative core beliefs play a crucial role in the development of psychosis, often overlapping with symptoms like voices and paranoia and being shaped by trauma and negative social experiences, (iii) a synergistic relationship between core beliefs and symptoms highlights how negative self-beliefs significantly affect symptoms, with voices often reinforcing these beliefs, (iv) core beliefs associated with images, and their influence on psychosis symptoms.  This study provides new insights into the range and character of core beliefs in psychosis and provides important data to guide ongoing and future development of treatment approaches for psychosis. |
| Taylor et al. (2020b) | UK  Cross-sectional study | 5 SZ (PD) | 23.4 (6.42) | Psychotic Symptom Rating Scales (PSYRATS)Positive Negative Syndrome Scale (PANSS) Brief Core Schema Scales (BCSS) Young Schema Questionnaire-Short Form (YSQ-S) | Results for the iMAgery focused psychological therapy for persecutory delusions in PSychosis (iMAPS) (6 session intervention) demonstrated significant reductions in negative schematic beliefs, delusions, and imagery distress.  iMAPS appears a feasible and acceptable treatment for psychosis, and further evaluation is indicated. |
| Thomas et al. (2015) | Australia  Cross-sectional study | 34 SZ (22M/12F) Outpatient | 35.4 (8.52) | Positive and Negative Syndrome Scale (PANSS)  Schedule for the Assessment of Insight (SAI)  Psychotic Symptom Rating Scales (PSYRATS)  Questionnaire-Revised (BAVQ-R)  Brief Core Schema Scales (BCSS) | Beliefs about voices were correlated with both negative voice content and schemas. After controlling for negative voice content, schemas were estimated to predict between 9% and 35% of variance in the six beliefs about voices that were measured. Negative-self schemas were the strongest predictors, and positive-self and negative-other schemas also showed potential relationships with beliefs about voices.  Schemas, particularly those regarding the self, are potentially important in the formation of a range of clinically relevant beliefs about voices. |
| Üçok et al. (2024) | Turkey, Netherlands,  USA,  UK Cross-sectional study | 742 SZ  (495M/247F)  718 SB  (335M/383F)  1039 HC (472M/567F) Inpatient & Outpatient | SZ 33.13 (8.3) SB 33.31 (9.3) HC 33.82 (11) | Childhood Trauma Questionnaire (CTQ) Brief Core Schema Scale (BCSS)   Operational Criteria for Psychotic Illness (OPCRIT) Structured Interview for Schizotypy–Revised (SIS-R) | Childhood Trauma was related to negative cognitive schemas toward self/others in Schizophrenia patients (SZ), siblings (SB), and controls (HC). Negative self-schemas mediated the relationship between emotional abuse and thought withdrawal and thought broadcasting. Approximately 33.9% of the variance in these symptoms was explained by this mediator. It also mediated the relationship between sexual abuse and persecutory delusions in SZ. SZ patients scored higher on negative other beliefs compared to SIB and HCs (p<0.001). SZ also scored lower on positive self-beliefs, compared to SIB and HCs (p<0.001). Childhood trauma was more prevalent in the SZ group across all types: physical abuse (63% vs. 17% SIB, 11.1% HC), sexual abuse (32.6% vs. 15.9% SIB, 9.8% HC), emotional abuse (43.3% vs. 22.1% SIB, 14.8% HC), physical neglect (58.2% vs. 44% SIB, 39.5% HC), and emotional neglect (55.2% vs. 42.9% SIB, 34% HC).  These findings suggest that childhood abuse and neglect are more common in patients with schizophrenia than their siblings and healthy controls, and have different impacts on clinical domains examined. The relationship between CT and positive symptoms seems to be mediated by negative cognitive schemas about self in schizophrenia. |
| Vorontsova et al. (2013) | UK  Cross-sectional study | 60 PD (19M/41F) 30 PD+D (17M/13F)  30 D (14M/16F)  30 HC (13M/17F) Outpatients | PD 40.1 (10.7) PD+D 42.8 (9.6)  D 42.5 (13.1)  HC 40.4 (13.1) | Schedules for Clinical Assessment in Neuropsychiatry (SCAN v2.1) Psychotic Symptom Rating Scales (PSYRATS) Green et al. Paranoid Thoughts Scale (GPTS) Brief Core Schema Scales (BCSS) | The results indicated that the Persecutory Delusion (PD) + Depression (D) group scored significantly higher on negative self-beliefs (p<0.01) and lower on positive self-beliefs (p<0.01) than the PD group. The Depression group scored significantly higher on both negative subscales and lower on both positive subscales than the HC group (all p<0.01).   These findings support the relevance of current clinical research trials targeting beliefs about the self and worry in people with delusions. Translation of further techniques from depression treatment for use with people experiencing psychosis is indicated. |
| Waite et al. (2019) | UK  Cross-sectional study | 60 SZ VH (36M/24F) | 41.9 years (11.7) | Body-Esteem Scale for Adolescents and Adults (BESAA) BMI Cardiff Anomalous Perceptions Scale (CAPS) hallucinations subscale Green et al Paranoid Thoughts Scale (GPTS) Brief Core Schema Scales (BCSS) Beck Depression Inventory-II (BDI) Penn State Worry Questionnaire (PSWQ) | Negative self-beliefs were correlated with Total Negative Voice Content (r=0.473, p<0.001), and Positive Voice Content (r=-0.384, p=0.002). Positive self-beliefs were correlated with Total Negative Voice Content (r=-0.321, p=0.012) and Total Positive Voice Content (r=0.262, p=0.043). Negative voice content regarding appearance was positively associated with negative beliefs about the self (medium effect size) and negatively associated with positive beliefs about the self (medium effect size). Conversely, positive voice content regarding appearance was positively associated with positive beliefs (small effect size) about the self and negatively with negative beliefs about the self (medium effect size).   Negative voice content about appearance appears to reflect negative self-beliefs. |

*Key: ARMS, At-risk Mental State; AVH, Auditory Verbal Hallucinations; BD, Bipolar Disorder; BPD, Borderline Personality Disorder; CAARMS, Comprehensive Assessment of At-Risk Mental States; CAMHS, Child and Adolescent Mental Health Services; CHR, Clinical High-risk; D, Depression; DEP, Depression; F, Female; HC, Healthy Controls; M, Male; MDD, Major Depressive Disorder; MH, Mental Health; OCD, Obsessive-compulsive Disorder; PD, Persecutory Delusions; PE, Psychotic Experiences; PLE/PLES, Psychotic-like Experiences; PNS, Persistent Negative Symptoms; PPS, Persistent Positive Symptoms; SB, Siblings; SSD, Schizophrenia-spectrum Disorder; SZ, Schizophrenia; SZA, Schizoaffective Disorder; UES, Unusual Experiences; UHR, Ultra-high risk; VH, Voice Hallucinations.*

104. Fowler, D., Freeman, et al. The Brief Core Schema Scales (BCSS): psychometric properties and associations with paranoia and grandiosity in non-clinical and psychosis samples. *Psychological medicine*, **36**(6), 749–759 (2006).

105. Fowler, D.  et al. Negative cognition, depressed mood, and paranoia: a longitudinal pathway analysis using structural equation modeling. *Schizophrenia bulletin*, **38**(5), 1063–1073 (2012).

106. Freeman, D. et al. The Stress of the Street for Patients With Persecutory Delusions: A Test of the Symptomatic and Psychological Effects of Going Outside Into a Busy Urban Area. *Schizophrenia bulletin*, **41**(4), 971–979 (2015).

107. Monsonet, M. et al. Self-Schemas and Self-Esteem Discrepancies in Subclinical Paranoia: The Essential Role of Depressive Symptoms. *Frontiers in psychiatry*, **12,** 623755 (2021).

108. Saleem, M. M. et al. Perceived discrimination in those at clinical high risk for psychosis. *Early intervention in psychiatry*, **8**(1), 77–81 (2014).

109. Shahravan, N., Rezaei, O., & Hosseini, R. The relationship between delusional beliefs and early maladaptive schemas in psychotic patients. *GMP Rev*, **18,** 3 (2015).

110. Smith, B. et al. Emotion and psychosis: links between depression, self-esteem, negative schematic beliefs and delusions and hallucinations. *Schizophrenia research*, **86**(1-3), 181–188 (2006).

111. Stowkowy, J., & Addington, J. Maladaptive schemas as a mediator between social defeat and positive symptoms in young people at clinical high risk for psychosis. *Early intervention in psychiatry*, **6**(1), 87–90 (2012).

112. Taylor, C. D., & Harper, S. F. Early maladaptive schema, social functioning and distress in psychosis: A preliminary investigation. *Clinical Psychologist*, **21**(2), 135-142 (2017).
