# Supplementary material for: Core beliefs in psychosis: a systematic review and meta-analysis
Source: Schizophrenia (Heidelb). 2025 Mar 6;11(1):38. doi: 10.1038/s41537-025-00577-2 (PMC11885481; doi:10.1038/s41537-025-00577-2)
Supplement: Supplementary file 4 — Appendix 4 [file 41537_2025_577_MOESM4_ESM.docx]

**APPENDIX 4: Characteristics of CHR Sample Studies (n=22)**

| **Author (Year)** | **Country & Type of Study** | **Sample Size & Setting** | **Mean Age (SD)** | **Questionnaires & Diagnostic Tools** | **Main Findings & Clinical Implications** |
| --- | --- | --- | --- | --- | --- |
| Addington & Tran (2009) | Canada  Cross-sectional study | 38 CHR  (28M/10F) Outpatient | 19.7 (3.4) | Brief Core Schema Scale (BCSS) Young Schema Questionnaire (YSQ-S)  The Scale of Prodromal Symptoms (SOPS) Calgary Depression Scale for Schizophrenia (CDSS) | Unusual thought content was positively correlated with negative self-schemas (r=0.41, p<0.05) and negative other schemas (r=0.44, p<0.01). Suspiciousness was correlated with negative self-schemas (r=0.39, p<0.05) and negative other schemas (r=0.67, p<0.01). The total positive symptom score was positively correlated with negative self-schemas (r=0.47, p<0.01) and negative other schemas (r=0.68, p<0.01). Low ratings on disorganised communication were negatively correlated to positive other schemas (r=–0.33, p<0.05). Perceptual abnormalities were positively correlated to negative other schemas (r=0.46, p<0.01).   The BCSS appears to be a valid scale for use in CHR to identify positive and negative self/other evaluations, which may be clinically meaningful intervention targets in psychosocial interventions. |
| Addington et al. (2013) | USA Cross-sectional study | 360 CHR  (210M/150F) 180 HC  (100M/80F) Outpatient | CHR 18.98 (4.18) HC 19.54 (4.78) | Childhood Trauma Questionnaire (CTQ) Psychosis Screening Questionnaire (PSQ) Brief Core Schema Scales (BCSS) | CHR participants experienced significantly more types of trauma (p<0.05) and bullying (p<0.05) compared to HCs. Negative self and other schemas were statistically significantly positively correlated with total trauma, and trauma subtypes.  These results offer preliminary support for an association between a history of trauma and later subthreshold symptoms. |
| Anilmis et al. (2015) | UK  Cross-sectional study | 94 CHR  (56M/38F)  Outpatient | 11.2 (1.9) | Unusual Experiences Questionnaire (UEQ) Bullying Questionnaire  Brief Core Schema Scale (BCSS) | Both negative self (NS) schemas and negative other (NO) schemas were associated with bullying (NS: r= 0.40, p <0.001; NO: r=0.33, p=0.002), and with Distressing Unusual Experiences (UEDs) (NS: r = 0.51, p< 0.001; NO: r =0 .43, p< 0.001). Both negative self-schemas and negative other schemas significantly mediated the relationship between bullying and UEDs (NS: z = 3.15, p= 0.002; NO: z = 2.35, p= 0.019).   Children’s negative self-beliefs may mediate the adverse psychological impact of victimisation and are appropriate treatment targets for young people with UEDs. Early educational intervention to reduce negative appraisals of the self and others may increase resilience to future adverse experiences and reduce later mental health risk. |
| Appiah-Kusi et al. (2017) | UK  Cross-sectional study | 30 UHR  (16M/14F)  38 HC  (18M/18F, 2 missing)  Outpatient | UHR 23.93 (4.78) HC 26.14 (4.69) | Childhood Trauma Questionnaire (CTQ)  Brief Core Schema Scale (BCSS)  Beck Depression Inventory  Paranoid Scales (PTS)  Psychosis Screening Questionnaire (PSQ) | UHR patients were significantly more likely to report exposure to various types of childhood trauma (emotional and sexual abuse, and emotional and physical neglect), had more negative schema and less positive schema about themselves and others, and were more likely to use cannabis more than once a month. Emotional neglect was found to be significantly associated with UHR status even after controlling for the effects of previous exposure to cannabis use (b=0.262, 95% CI: 0.115-0.408), and this association was partially mediated by negative self-schema (b=0.045, 95% CI: 0.004-0.159). Similarly, emotional neglect was significantly associated with paranoia (b = 1.354, 95% CI: 0.246-2.462), and this association was partially mediated by negative self-schema (b=0.988, 95% CI: 0.323-1.895).  These results suggest that interventions to modify negative schema may be a potential treatment approach in individuals presenting with ultra-high risk for psychosis particularly those who have a history of emotional neglect in childhood. |
| Bird et al. (2017) | UK  Longitudinal study  Self-report & interview assessments at baseline & 3-month follow-up | 34 ARMS  (6M/28F) Outpatient | 14.9 (1.25) | The Green et al. Paranoid Thoughts Scale (GPTS)   The Penn State Worry Questionnaire for Children (PSWQ-C)  Brief Core Schema Scales (BCSS)  The Perceived Stress Scale (PSS) Cyber Victim and Bullying Scale (CVBS)  Rosenberg Self-Esteem Scale (RSES) | Significant predictors of paranoia persistence were anxiety, depression, worry, negative self-beliefs, perceptual anomalies, insomnia, affective reactivity, bullying, and cyber victimization. Heightened perceived stress (r=0.65, p<0.001), negative affect on social media (r=0.53, p=0.002), peer bullying (r=0.59, p<0.001), and cyber victimization (r=0.39 p<0.001) significantly predicted paranoia persistence after three months with medium to large effect sizes. Negative self-schemas significantly correlated with paranoia severity at baseline (r=0.51, p<0.002), and at 3- month follow up (r=0.57, p<0.001).   Assessment and screening of paranoia in adolescent mental health services may therefore be important for effective early intervention. Second, with the majority of participants reporting persistent persecutory ideas the identification of clear maintenance factors provides potential avenues for treatment development. |
| Braun et al. (2022) | USA  Cross-sectional study | 203 CHR  (99M/104F)  Outpatient | 17.4 (3.9) | Brief Core Schema Scale (BCSS)  Defeatist Performance Attitude Scale (DPAS) Asocial Beliefs Scale (ABS)  First Episode Social Functioning Scale (FESFS) | Of their CHR Sample, 72.9% reported experiencing bullying. These participants had greater severity of negative schemas about others and asocial and defeatist performance beliefs, and lower social functioning scores. Negative other schemas were statistically higher in the Bullied group compared to the Non-bullied group (p<0.05). Bullying was positively correlated with negative self-schemas (r=0.20, p<0.05).   Prevalence of bullying among CHR participants is high. Bullying may be a risk factor for increased asocial and defeatist beliefs, negative core schemas, and poor social functioning. Targeting maladaptive schemas and beliefs during treatment may serve to improve functional outcomes in this group. |
| Cowan et al. (2019) | USA  Cross-sectional study | 73 UHR  (44M/29F)  73 HC  (32M/41F)  Outpatient | UHR 18.7 (1.8)  HC 18.1 (2.6) | Brief Core Schema Scales (BCSS) Beck Depression Inventory—II (BDI) Beck Anxiety Inventory (BAI) Structured Interview for Prodromal Symptoms (SIPS) Structured Clinical Interview for DSM-IV Axis I Disorders (SCID) | UHR youth reported significantly more negative beliefs about self and others, and significantly less positive beliefs about self and others. HC youth rarely endorsed negative self-beliefs. UHR youths’ core beliefs were linked to attenuated psychotic symptoms (particularly negative symptoms and suspiciousness) and depressive symptoms, but not anxiety symptoms.    This study contributes to a growing literature on cognitive factors in youth at high risk for psychotic disorders. The findings that UHR reports higher negative and fewer positive beliefs about self and others suggest that early identification and intervention targeting core beliefs could be crucial. CBT may focus on modifying negative self-schemas to prevent FEP and lower levels of depression in UHR populations. |
| Cowan et al. (2024) | USA Cross-sectional study | 59 CHR  (22M/27F) 52 HC (33M/19F)  Outpatient | CHR 20.86 (2.78) HC 21.87 (3.47) | Rosenberg Self-Esteem Scale (RSES) Brief Core Schema Scales (BCSS) Self-concept Clarity Scale (SCS) Rumination and Reflection Questionnaire (RRQ) Structured Interview for Psychosis-Risk Syndromes (SIPS) Global Functioning Scales (CFS) | CHR participants reported more negative self-esteem and self-schemas, poorer self-concept clarity, and more ruminative self-focus, all of which related to negative symptoms. CHR participants narrated their life stories with themes of negative emotion and passivity which related to positive and negative symptoms. There were significant group differences in negative self-schemas (p<0.001), positive self-schemas (p=0.035), self-esteem (p=0.008), and self-concept clarity (p=0.002). Correlations in the CHR group included negative self-beliefs with negative symptoms (r=0.54, p<0.001), and self-esteem with social functioning (r=0.43, p=0.014).  This group of youth at CHR exhibited some, but not all, changes to self-concept and narrative identity seen in psychotic disorders. A core theme of negativity, uncertainty, and passivity ran through their semantic and narrative self-representations. Preserved self-reflection and auto-biographical reasoning suggest sources of resilience and potential footholds for cognitive-behavioural and metacognitive interventions. |
| Crowter et al. (2022) | UK Cross-sectional study | 133 ARMS  137 non-ARMS (55%M/45%F)  Outpatient | 19.5 (2.6) Total | Comprehensive Assessment of At-Risk Mental States (CAARMS) Brief Core Schema Scales (BCSS) Beck Depression Inventory-II (BDI-II) Social Interaction Anxiety Scale (SIAS) Schizotypal Symptoms Inventory – Brief Version (SSI) | The ARMS group displayed significantly higher negative self and negative other schemas compared to the non-ARMS group (p<0.001). The ARMS group also endorsed a significantly lower positive-other schema in comparison with participants in the non-ARMS group, although there were no significant group differences for positive self-schema. Depression, social anxiety, and negative-self and -other schema all significantly predicted higher scores on the paranoia subscale of the SSI. Of the schema subscales, negative self-beliefs predicted higher levels of both depression and social anxiety. Furthermore, negative-other schema was associated with depression. Positive-other schema had no direct relationship with paranoia but was predictive of lower social anxiety.  The presence of negative-self and other schematic beliefs, rather than the absence of positive schematic beliefs, may be a potential therapeutic target for at-risk young people experiencing paranoia. |
| Devoe et al. (2021) | USA,  Canada Cross-sectional study | 740 CHR Total  67 Persistent Negative Symptoms (PNS) (48M/19F)  673 non-PNS  (376M/297F) Inpatient & outpatient | PNS 18.7 (4.04) non-PNS 18.5 (4.28) | Scale of Psychosis-risk Symptoms (SOPS)  Brief Core Schema Scale (BCSS) Presence of Psychotic Symptoms (POPS) Calgary Depression Scale for Schizophrenia (CDSS) | Compared to the non-PNS group, the Persistent Negative Symptom (PNS) participants had significantly higher levels of total negative-self schemas at 12-months (p<0.05) and 24-months (p<0.05).The non-PNS group significantly improved on total negative-self schemas at all time points compared to baseline, while the PNS group only significantly improved on total negative-self schemas at 18- months compared to baseline.  PNS exist in youth at CHR for psychosis, resulting in significant and persistent functional impairment, which remains when controlling for persistent depressive symptoms. PNS remain even in CHR youth who do not transition to psychosis. Thus, PNS may represent an unmet therapeutic need in CHR populations for which there are currently no effective treatments. |
| Devoe et al. (2022) | USA  Cross-sectional study | 203 CHR  (99M/104F) Outpatient | 17.4 (3.9) | Brief Core Schema Scale (BCSS) Structured Interview for Psychosis-risk Syndromes (SIPS) Scale of Psychosis-risk Symptoms (SOPS) Defeatist Performance Attitude Scale (DPAS) Asocial Beliefs Scale (ABS) | Negative self-beliefs were associated with negative symptoms (r=0.182, P<0.05) and asocial beliefs (r=0.402, p<0.01). Defeatist performance attitudes were associated with negative self (r=0.636, p<0.01) and negative other beliefs (r=0.341, p<0.01).  Negative symptoms are common in individuals at CHR for psychosis, and beliefs and attitudes may play an important role in the relationship between poor functioning and severity of negative symptoms. Thus, psychosocial interventions may wish to target beliefs and attitudes in an effort to reduce negative symptoms in CHR youth. |
| Gin et al.  (2021) | UK Cross-sectional study | 122 CHR  (76.2%F/27.8%M)  Outpatient | 14.81 (1.62) | Unusual Experiences Questionnaire (UEQ) Brief Core Schema Scale (BCSS) Life Events Questionnaire and Brief Trauma Checklist Adolescent version of the Dissociative Experiences Scale (A-DES) Difficulties in Emotion Regulation Scale (DERS) | The psychological mechanisms of adult cognitive models of psychosis explained 89% of the total variance of adolescent Distressing Unusual Experiences (UED) severity (p<0.0005), with schemas as the principal significant contributor. Variance explained 40-72% across each of the UE types (paranoia, hallucinations, delusions, paranormal thinking and grandiosity). UED severity was correlated with negative self-schemas (r=0.256, p<0.01) and negative other schemas (r=0.246, p<0.01), positive other schemas (r=-0.180, p<0.05), and difficulties in emotion regulation (0.289, p<0.01) indicating that higher levels of negative self-schemas are associated with greater severity of distressing unusual experiences.  Findings suggest that the psychological components of adult cognitive models of psychosis, particularly schemas, are also implicated in adolescent distressing unusual experiences. |
| LoPilato et al. (2021) | USA,  Canada  Cross-sectional study | 531 CHR  (321M/210F) Outpatient | 18.80 (4.21) | Childhood Adversity: Childhood Trauma and Abuse scale Cognitive Schemas: Brief Core Schema Scale (BCSS) Attenuated Positive Symptoms: Structured Interview for Prodromal Syndromes (SIPS) Depression: Calgary Depression Scale for Schizophrenia (CDSS) | There were no direct effects of either threat or deprivation on any of the attenuated positive psychotic symptom domains. There was a direct effect of threat, but not deprivation, on negative-other and negative-self schemas. Negative-other schemas had a direct effect on delusional thinking and suspiciousness. There was a significant indirect effect of threat on delusional thinking and suspiciousness through negative-other schemas. Negative-self schemas had a direct effect on delusional thinking; however, there were no significant indirect effects. Finally, there were no direct effects of total adversity on any of the attenuated positive psychotic symptoms, however there was a significant indirect effect of total adversity on delusional thinking and suspiciousness through negative-other schemas.  Cognitive vulnerability in the form of negative schemas about others may be one mechanism linking childhood threat experiences and attenuated psychotic symptoms. The results underscore the importance of targeting negative schemas in interventions to mitigate psychosis risk. |
| Marshall et al. (2016) | Canada,  USA Cross-sectional study | 442 CHR in total 213 With violent content  (122M/91F) 229 Without violent content  (134M/95F) Outpatient | With violent content 18.75 (4.16)  Without violent content 18.74 (4.17) | Structured Interview for Prodromal Syndromes (SIPS) Scale of Prodromal Symptoms (SOPS) Calgary Depression Scale for Schizophrenia (CDSS) Brief Core Schema Scales (BCSS) Abuse/Trauma Questionnaire CAPS (Content of Attenuated Positive Symptoms Codebook) | Individuals with violent content had significantly higher APS, greater negative beliefs about the self and others, and increased bullying. The same findings and higher ratings on anxiety symptoms were present when participants with self-directed violence were compared to participants with no violent content. Individuals reporting violent content differ in their clinical presentation compared to those who do not experience violent content. Adverse life events, like bullying, may impact the presence of violent content in APS symptoms. Future studies should explore violent content in relation to actual behaviour.  Based on the current study, it may be worthwhile for clinicians to pay attention to specific symptom content that is presented in attenuated psychotic symptoms. Specific content may indicate the presence of other clinical and cognitive profiles, such as increased attenuated psychotic symptoms or anxiety and more negative beliefs about themselves and/or other people. The current findings further highlight the significance of clinicians asking about self-directed violence above and beyond self-harm and suicidal ideations. Being aware of symptom content may give clinicians more information about potential safety concerns. |
| Marulanda & Addington (2016) | Canada  Cross-sectional study | 40 CHR  (22M/18F) 40 HC  (21M/19F) Outpatient | CHR 17.05 (2.69) HC 19.13 (1.36) | Structured Interview for Prodromal Syndromes (SIPS) Connor–Davidson Resilience Scale (CD-RISC) Child and Youth Resilience Measure (CYRM) Calgary Depression Scale for Schizophrenia (CDSS) Social Interaction Anxiety Scale (SIAS) Brief Core Schema Scale (BCSS) Global Functioning Scale (Social and Role) Childhood Trauma and Abuse Scale | Significant difference between the groups on levels of resilience (p<0.01) demonstrating that CHR participants have lower levels of resilience than HCs. In terms of the associations between resilience and measures of mental health of CHR participants, it was found that higher levels of resilience were related to lower negative symptoms, depression and anxiety. Furthermore, resilient CHR participants showed higher levels of role functioning and generally reported higher positive schemas of self and others, as well as lower stress to reported life events. No associations were found between resilience and attenuated psychotic symptoms, social functioning, IQ and trauma.  Interventions aimed at promoting resilience can be helpful in improving the functioning of CHR individuals in society by decreasing feelings of anxiety, depression and negative symptoms, promoting positive beliefs of self and others, and reducing stress reactivity to life events. Furthermore, resilience interventions, particularly those that promote social support, can also have a positive impact on mental health by encouraging health-seeking behaviours. |
| Michaels et al. (2023) | USA Cross-sectional study | 686 CHR  (393M/293F)  252 HC  (130M/122F) Outpatient | CHR 18.53 (4.29)  HC 19.87 (4.67) | Perceived Discrimination (PD) Scale Brief Core Schema Scale (BCSS) Structured Clinical Interview for Psychosis-Risk Syndromes (SIPS) Scale for the Psychosis-Risk Symptoms (SOPS) | CHR individuals report higher levels of past year and lifetime Perceived Discrimination (PD) compared to HCs. Lifetime ethnoracial PD was associated with suspiciousness and total positive symptoms. Negative schema of self and others scores partially mediated the relation of past year ethnoracial PD to suspiciousness, one of five positive symptom criteria for CHR. For CHR individuals, past year ethnoracial discrimination was associated with negative beliefs about themselves and others, which was associated with suspiciousness.   These findings contribute to an emerging literature characterizing the mechanisms by which discrimination contributes to the positive symptoms characterizing the CHR syndrome. |
| Morrison et al. (2015) | UK Cross-sectional study | 117 CHR  (60.8% M/39.2%F) Outpatient | 20.3 (SD not specified) | Comprehensive Assessment for At Risk Mental States (CAARMS) Beck Depression Inventory for Primary Care (BDI-PC) Beliefs About Paranoia Scale (BAPS) Brief Core Schema Scale (BCSS) Persecution and Deservedness Scale (PaDS) | The study found that the sample of at-risk mental state participants was not as paranoid but reported higher levels of ‘bad-me’ deservedness, compared with psychiatric in-patients. Negative self-schemas were related to deservedness but not paranoia, whereas negative beliefs about others were positively related to paranoia but negatively with deservedness. Both depression and negative metacognitive beliefs about paranoid thinking were specifically related to paranoia but not deservedness. Results showed significant correlations between the Persecution and Deservedness Scale and negative other schemas (r=0.57, p<0.01), negative self-schemas (r=0.47, p<0.01).  This study provides evidence for the role of negative cognition, metacognition and negative affect in the development of paranoid beliefs, which has implications for psychological interventions and our understanding of psychosis. |
| Müller et al. (2018) | Germany  Cross-sectional study | 348 total 137 CHR (92M/45F) 211 PPS (Persisting Positive Symptoms) (128M/83F)  Outpatient | CHR 25.1 (5.3) PPS 37.5 (9.8) | Positive and Negative Syndrome Scale (PANSS) Structured Interview for Prodromal Syndromes (SIPS) Schizophrenia Proneness Instrument-Adult Version (SPI-A) Brief Core Schema Scale (BCSS) | In the CHR sample, there were correlations between PANNS persecution and: negative self-schemas (r=0.298, p<0.001), positive self-schemas (r=−0.191, p=0.026), negative other schemas (r=0.189, p=0.028), and positive other schemas (r=−0.254, p=0.003). In the PPS sample, there was an association between PANSS persecution and: negative self-schemas (r=0.182, p=0.008) and negative other schemas (r=0.348, p<0.001). In the overall CHR sample as well as in the lower CHR sub-class III, paranoid ideation is associated with negative beliefs about the self. On the other hand, negative beliefs about others are associated with paranoid ideation in the highest risk states and full-blown psychosis.  The presented results support schema-based diagnostic efforts and psychotherapy in patients at CHR with high suspiciousness scores in early detection and intervention centres and in individuals with persecutory delusions within clinical settings. |
| Patton et al. (2022) | USA Longitudinal study, Baseline & 12 month follow up | 73 CHR (at baseline) 54 CHR (at 12-month follow-up)  Outpatient | 18.6 (1.8) | Brief Core Schemas Scale (BCSS) Structured Interview for Prodromal Symptoms (SIPS) Global Functioning Scale (GFS) Beck Depression Inventory-II (BDI-II) | Longitudinal changes in self-beliefs within a CHR sample were associated with symptoms and functioning at 12-month follow-up. Although baseline self-beliefs did not add predictive validity when controlling for depression, changes in self-beliefs from baseline to follow-up predicted more negative symptoms and worsening social and role functioning at follow-up. Notably, the independent effect of an increase in negative self-beliefs marginally or significantly predicted all outcomes of interest.  Results suggest that schema-focused therapy may be a useful intervention for CHR populations and 12 months would be a meaningful amount of time in which to administer therapy in the dynamic late adolescent/early adult developmental period. Moreover, as maladaptive self-schemas have been linked to early childhood maltreatment, these interventions might help to buffer the effects of earlier adverse experiences. |
| Saleem et al. (2014) | USA,  Canada Cross-sectional study | 360 CHR  (211M/149F) 180 HC  (87M/93F) | CHR 18.99 (4.18)  HC 19.54 (4.77) | Scale of Prodromal Symptoms (SOPS) Brief Core Schema Scale (BCSS) Structured Interview for Prodromal Syndromes (SIPS) Self-report measure of perceived discrimination | CHR participants reported significantly more perceived discrimination. Perceived discrimination was significantly associated with negative schemas, but not with attenuated positive symptoms. CHR participants reported significantly higher levels of negative schemas about the self (U = 196.23, P < 0.0001) and about other people (U =136.04, P < 0.0001) compared with HC.  These results suggest that individuals at CHR for psychosis endorse a higher level of perceived discrimination, which is associated with increased negative schemas, but not attenuated positive symptoms. |
| Stowkowy & Addington (2012) | Canada Cross-sectional study | 38 CHR  (28M/10F)  23 HC  Outpatient | CHR 19.7 (3.4)  HC (age matched) | Structured Interview for Prodromal Symptoms (SIPS) Scale for Assessment of Prodromal Symptoms (SOPS) Calgary Depression Scale Brief Core Schema Scales (BCSS) Social Defeat Scale | Compared with HCs, CHR participants evidenced higher levels of total negative beliefs (p<0.0001). The study found that Social Defeat was statistically significantly higher in CHR than in the HCs (p<0.01). Overall, results found high levels of social defeat and negative self and other schemas in the CHR population. Negative beliefs mediated the relationship between social defeat and early symptoms, offering some support for the notion that maladaptive self-schemas play a role in the onset of psychosis.  These results have implications for prevention because these maladaptive schemas are malleable factors for which we have effective psychological interventions. |
| Stowkowy et al. (2016) | USA,  Canada  Cross-sectional study | 765 CHR  (436M/329F)  280 HC  (141M/139F) Outpatient | CHR 18.47 (4.24)  HC 19.65 (4.67) | Structured Interview for Prodromal Syndromes (SIPS) Scale for Assessment of Prodromal Symptoms (SOPS) Brief Core Schema Scale (BCSS) Calgary Depression Scale for Schizophrenia (CDSS) Childhood Trauma and Abuse Scale | CHR participants evidenced stable and more maladaptive schemas over time compared to controls. Schemas at initial contact did not vary amongst the different clinical outcome groups at 2 years although all CHR outcome groups evidenced significantly worse schemas than healthy controls. Although there were no differences on baseline schemas between those who later transitioned to psychosis compared to those who did not, those who transitioned to psychosis had more maladaptive negative self-schemas at the time of transition. After controlling for depression, negative self-beliefs were correlated with physical abuse (r= 0.13, p<0.001) and psychological abuse (r=0.16, p<0.001), while negative other beliefs were correlated with psychological bullying (r=0.17, p<0.001) and total positive symptoms of psychosis (r=0.23, p<0.001).  These findings demonstrate a need for interventions that aim to improve maladaptive schemas among the CHR population. Therapies targeting self-esteem, as well as schema therapy may be important work for future studies. |

*Key: ARMS, At-risk Mental State; AVH, Auditory Verbal Hallucinations; BD, Bipolar Disorder; BPD, Borderline Personality Disorder; CAARMS, Comprehensive Assessment of At-Risk Mental States; CAMHS, Child and Adolescent Mental Health Services; CHR, Clinical High-risk; D, Depression; DEP, Depression; F, Female; HC, Healthy Controls; M, Male; MDD, Major Depressive Disorder; MH, Mental Health; OCD, Obsessive-compulsive Disorder; PD, Persecutory Delusions; PE, Psychotic Experiences; PLE/PLES, Psychotic-like Experiences; PNS, Persistent Negative Symptoms; PPS, Persistent Positive Symptoms; SB, Siblings; SSD, Schizophrenia-spectrum Disorder; SZ, Schizophrenia; SZA, Schizoaffective Disorder; UES, Unusual Experiences; UHR, Ultra-high risk; VH, Voice Hallucinations.*
