# Supplementary material for: Core beliefs in psychosis: a systematic review and meta-analysis
Source: Schizophrenia (Heidelb). 2025 Mar 6;11(1):38. doi: 10.1038/s41537-025-00577-2 (PMC11885481; doi:10.1038/s41537-025-00577-2)
Supplement: Supplementary file 5 — Appendix 5 [file 41537_2025_577_MOESM5_ESM.docx]

**APPENDIX 5: Characteristics of the Non-Clinical Samples Studies (n=14)**

| **Author (Year)** | **Country & Type of Study** | **Sample Size & Setting** | **Mean Age (SD)** | **Questionnaires & Diagnostic Tools** | **Main Findings & Clinical Implications** |
| --- | --- | --- | --- | --- | --- |
| Ashford et al. (2012) | UK  Cross-sectional study | 135 Non-Clinical Sample  (12M/123F)  Outpatient | 19.8 (unspecified) | Modified Direct and Indirect Aggression Scales (DIAS) Interpersonal Sensitivity Measure (IPSM)  Hospital Anxiety and Depression Scale (HADS) Brief Core Schema Scales (BCSS) Green et al. Paranoid Thought Scales (GPTS) | Negative self-beliefs and depression significantly mediated the relationship between indirect aggression and paranoid thinking, whereas negative beliefs about others mediated the relationship between direct verbal aggression and paranoid thinking. These findings suggest that negative beliefs and depression are the mediators of the relationship between bullying and paranoid thinking thus further contributing to the understanding of the association between adverse early life experiences and paranoid thinking.   This study has provided some evidence that negative self-beliefs and depression mediate the relationship between bullying experienced in childhood and paranoid thinking in adulthood in a non-clinical population. |
| Barrantes-Vidal et al. (2013) | Spain, USA  Cross-sectional study | 214 Non-Clinical Sample  (22%M/78%F) Outpatient | 21.4 (2.4) | Wisconsin Schizotypy Scales (WSS) Community Assessment of Psychic Experiences (CAPE) Schizotypal Personality Questionnaire (SPQ) Brief Core Schema Scales (BCSS) Calgary Depression Scale Beck Depression Inventory-II (BDI-II) Rosenberg Self-Esteem Scale | Positive schizotypy was associated with measures of anxiety and depression, and with low self-esteem and negative self and other schemas. In contrast, negative schizotypy was associated with diminished positive self and other schemas.   This pattern of results highlights the differential role of affect, such that positive schizotypy tends to be characterized by affect dysregulation and high negative affect, whereas negative schizotypy is associated with diminished positive affect. This distinction offers insight into the long-term trajectories of the dimensions, such as social anxiety for positive and schizoid withdrawal for negative schizotypy and has the potential to isolate specific mechanisms that can be targeted with treatment interventions. |
| Bennetts et al. (2020) | UK  Cross-sectional study | 24 Non-Clinical Sample students with high levels of non-clinical paranoia (21%M/79%F) Outpatient | 20.54 (4.93) | Adapted Paranoia Checklist Paranoia Scale State and Trait Anxiety Inventory Brief Core Schema Scales (BCSS) | Positive/safe interpersonal imagery reduced state and trait paranoia, and trait anxiety, and increased positive self-beliefs. Negative/threat imagery increased state paranoia, and state and trait anxiety. Neutral imagery reduced positive other beliefs.  Rehearsing interpersonal imagery in which the person experiences themselves as safe, secure, and able to trust others, may have large, sustained effects. A fully powered clinical study is warranted. |
| Bortolon et al. (2017) | France  Cross-sectional study | 425 Non-Clinical Sample  (363F/62M) Outpatient | 36.23 (15.2) | The Young Schema Questionnaire Short Form (YSQ-SF)  Childhood Trauma Questionnaire (CTQ)  The Dissociative Experiences Scale (DES)  Launay-Slade Hallucination Scale (LSHS-R) | Sexual and emotional abuse impact on AH both through the effect of maladaptive schemas and dissociation; (2) physical abuse impact on AH only through the effect of dissociation. These authors found that four maladaptive schemas impact on AH: Abandonment, Vulnerability, Self-sacrifice and Subjugation.  Overall, these findings indicate that specific early maladaptive schemas may play a fundamental role in the association between exposure to trauma and auditory hallucination together with dissociation symptoms in the general population. Consequently, the study suggests considering maladaptive schemas as an important therapeutic target when working with individuals experiencing AH with or without a psychiatry disorder. |
| Boyda et al. (2018) | USA  Cross-sectional study | 302 Non-Clinical Sample  (91M/211F) Outpatient | 36 (11.6) | Adverse Childhood Experiences International Questionnaire (ACE-IQ) Young’s Schema Questionnaire-Short Version (YSQ-SF) Community Assessment of Psychic Experiences (CAPE) | Emotional abuse was statistically associated with the following schemas: Defectiveness (p=0.012), Dependency (p=0.016) and Enmeshment (p=0.003). Sexual abuse showed a significant positive association with Defectiveness (p=0.013), Dependency (p<0.001) and Enmeshment (p<0.001). Sexual abuse was significantly negatively associated with emotional inhibition (p=0.014). There was a significant negative association between Emotional Inhibition and psychotic experiences (p<0.05). Results indicated specificity effects in that specific types of maltreatment are associated with specific maladaptive schemas.  These findings offer credence to cognitive theories of psychopathology and support the validity of EMS identification and modification among clients with psychotic symptomology both as a fundamental component of traditional CBT and within specialized schema‐focused therapy. |
| Chau et al. (2023) | China  Cross-sectional study | 134 Non-Clinical Sample  (41.8%M/58.2%F) Outpatient | 20.33 (2.91) | UCLA Loneliness Scale Version 3 (UCLA-LS-v3 Revised Green et al. Paranoid Thoughts Scale (R-GPTS) Social Interaction Anxiety Scale-6/Social Phobia Scale-6 (SIAS-6/SPS-6) Brief Core Schema Scale (BCSS) | Higher negative-self schema was associated with a stronger link from paranoia to social anxiety, whereas higher negative-other schema was associated with a stronger link from social anxiety to paranoia.   The findings support the reciprocal relationship between social anxiety and paranoia. While loneliness contributes to the development of social anxiety and paranoia, negative self and other schemas appear to modify the relationships between the two symptoms. |
| Fisher et al (2012) | UK  Cross-sectional study | 212 Non-Clinical Sample  (34.6%M/65.4%F) Outpatient | 27 (8.4) | Childhood Trauma Questionnaire (CTQ) Psychosis Screening Questionnaire (PSQ) Beck Depression Inventory (BDI) Beck Anxiety Inventory (BAI) Brief Core Schema Scales (BCSS) | Elevated rates of paranoia were only apparent amongst those reporting Emotional Abuse (present=50.9% vs. absent=27.0%; OR=3.26, 95% CI 1.56–6.81) and Physical Abuse (present=55.6% vs. absent=27.2%; OR=3.15, 95% CI 1.46–.80). The potential mediators were all significantly related to paranoia (anxiety: OR = 3.87, 95% CI 1.92–7.78; depression: OR=3.52, 95% CI 1.69–7.34; negative self-schemas OR=3.19, 95% CI 1.61– 6.33; negative other schemas OR=1.96, 95% CI 1.02–3.78).  Greater understanding of the pathways from childhood maltreatment to paranoia will enable suitable preventative interventions to be developed and targeted at those at highest risk. |
| Galbraith et al. (2014) | UK Cross-sectional study | Study 1  134 Non-Clinical Sample  (20M/114F)  Study 2  115 Non-Clinical Sample  (19M/96F)  Outpatient | Study 1: 21 years 7 months (5 years 5 months) Study 2: 22 years 10 months (6 years 3 months) | Persecutory Ideation Questionnaire (PIQ) Brief Core Schema Scales (BCSS) Depression Anxiety Stress Scales (DASS-21)  Social Avoidance and Distress Scale (SADS)  Rosenberg Self-Esteem Scale (RSES) | Negative self and other schemas significantly and positively predicted Persecutory Ideation (PI) (p<0.001, p<0.001, respectively). Anxiety partially mediated the relationship between negative self-schemas and PI but not negative other schemas and PI. Negative self-schemas, negative other schemas, and self-esteem predicted PI (p<0.01, p<0.001; p<0.001 respectively). Anxiety (p=0.001), and depression (p<0.001), but not social anxiety, (p=0.089) were all significant predictors of PI.  Targeting anxiety and depression may reduce the impact of self-schemas and low self-esteem on PI, but addressing negative affect alone may not necessarily reduce PI in those with preexisting negative other schemas. Additionally, therapists may help patients address both their self and other perceptions and develop awareness of how such schemas contribute to their persecutory beliefs. |
| Gibson et al. (2019) | USA  Cross-sectional study | 945 Non-Clinical Sample  (24.4M/75.6F) Outpatient | 20.13 (2.47) | Childhood Trauma Questionnaire (CTQ) Psychosis Screening Questionnaire (PSQ) Beck Depression Inventory (BDI) Beck Anxiety Inventory (BAI) Brief Core Schema Scales (BCSS) | This study found significant indirect effects from Traumatic Life Events (TLE) exposure to Psychotic-Like Experiences (PLEs) through perceived stress, dissociation, external locus of control, negative self-schemas, and negative other-schemas. When controlling for comorbid psychological symptoms, only the indirect effect from TLE exposure to PLEs through dissociation continued to be significant. Targeting stress sensitivity, maladaptive schemas, dissociative tendencies, and externalizing attributional styles may prove useful in the amelioration of risk for various psychopathologies (e.g., mood, psychosis) in the aftermath of TLE exposure. Negative self-schemas were significantly related to Traumatic life events (p<0.0001) and PLEs (r=0.36, p<0.0001), as were negative other schemas for TLEs (p<0.001) and PLEs (r=0.24, p<0.0001). Indirect effects indicated that greater perceived stress, dissociation, external locus of control, and negative self- and other schemas were significant putative mediators in the relationship between TLE exposure and higher PLE endorsement. When exploring the independent contribution of each mediator, all five variables statistically mediated the association between TLEs and PLEs.  Findings underscore the importance of targeting trauma-related cognitions in the prevention or reduction of psychotic-like experiences or disorders. |
| Gracie et al. (2007) | UK  Cross-sectional study | 228 Non-Clinical Sample  (67M/161F) Outpatient | 28.9 (8.7) | Traumatic Life Events Questionnaire (TLEQ) Self-Report Scale-Post Traumatic Stress Disorder (SRS-PTSD) Brief Core Schema Scale (BCSS) Paranoia Scale (PS) Launay Slade Hallucination Scale (LSHS) Structured Interview for Assessing Perceptual Anomalies (SIAPA) | Associations were found between negative schematic beliefs, PTSD and predisposition to both paranoia and hallucinations. PTSD re-experiencing symptoms were most strongly associated with a predisposition to hallucinations. Negative beliefs about self and others were most strongly associated with a predisposition to paranoia. Negative self-schemas and negative other schemas were correlated with paranoia (r= 0.54, p<0.0001, r=0.57, p<0.0001, respectively). Negative self-schemas and negative other schemas were correlated with hallucinations (r= 0.24, p<0.0001, r=0.33, p<0.0001, respectively). Positive self-beliefs and positive other beliefs were negatively correlated with paranoia (r=-0.42, p<0.0001, r=-0.48, p<0.0001, respectively) and hallucinations (r=-0.20, p<0.002, r=-0.15, p<0.02, respectively).  The results provide support for the prediction that there may be two routes between trauma and predisposition to psychosis. Clear support was found for a link between trauma and psychosis mediated by negative beliefs about self and others. There may also be a direct association between re-experiencing symptoms and hallucinations. |
| Monsonet et al. (2021) | Spain  Cross-sectional study | 208 Non-Clinical Sample of Undergraduates  Total  (22.1%M/77.9%F) 71 Control  34 Depression  41 Paranoia  32 Mixed Outpatient | 19.7 (2.3) | Implicit Self-Esteem: Go/No-Go Association Task (GNAT) Explicit Self-Esteem: Rosenberg Self-Esteem Scale (RSE) Brief Core Schema Scales (BCSS) Suspiciousness subscale of the Schizotypal Personality Questionnaire (SPQ-S) Depressive Symptoms: Beck Depression Inventory-II (BDI-II) | All groups presented similar and positive levels of implicit self-esteem. Trait-paranoia participants had similar levels of explicit self-esteem and self-schemas compared with the control group. However, the group with a combination of trait-paranoia and depressive symptoms showed the lowest levels of positive self-schemas and self-esteem. Furthermore, this group and the control group displayed implicit/explicit self-esteem discrepancies, although in opposite directions and with different implications. The dimensional approach revealed associations of trait-paranoia and depressive symptoms with poor explicit self-esteem and self-schemas but not with implicit self-esteem.  Trait-paranoia participants showed different self-representations depending on whether depressive symptoms were present or not. The interaction between subclinical neurotic and psychotic traits entailed a detrimental self-representation that might increase the risk for psychopathology. |
| Jaya et al. (2017) | Germany, Indonesia, USA Cross-sectional study | 2350 Non-Clinical Sample  786 Germany,  844 Indonesia,  720 USA  (62.2%M/37.8%F)  Outpatient | 32.53 (11.38) | Perceived Social Rank (PSR) Loneliness (LON) Community Assessment of Psychic Experiences (CAPE) Depression, Anxiety, and Stress Scale (DASS) Childhood Trauma Questionnaire (CTQ) Paranoia Checklist (PC) Brief Core Schema Scales (BCSS) Social Comparison Scale (SCS) | Social adversity had a significant medium to large effect on positive (r=0 .20) and negative symptoms (r2=0 .38). Social rank, negative schemas, and loneliness significantly mediated the relationship between social adversity and negative symptoms and the models explained a large amount of the variance (r=0.43–0.44). For positive symptoms, only negative schemas were a significant mediator (r= 0.27).   The results emphasize the role of social adversity in psychosis and support the assumption that cognitive vulnerability is a relevant translating mechanism as postulated by the social defeat hypothesis and cognitive models of psychosis. This underlines the relevance of the clinical practice of targeting beliefs in cognitive interventions for psychosis. It also indicates that targeting cognitive vulnerability in people experiencing social adversity could be a promising approach to prevention. |
| Sellers et al. (2018) | UK  Cross-sectional study | 227 Non-Clinical Sample  (40M/187F) Outpatient | 32.82 (13.36) | Paranoia Checklist (PCL) Metacognitions Questionnaire-30 (MCQ-30) Depression Anxiety Stress Scale-21 (DASS-21) Brief Core Schema Scale (BCSS) | The results demonstrated that unhelpful metacognitive beliefs had a positive moderating effect on the relationship between paranoia and negative affect. Negative beliefs about oneself and other people did not moderate negative affect but positive beliefs about other people had a negative moderating effect. In a final model, negative schematic beliefs predicted paranoid ideation whilst metacognitive beliefs predicted and moderated affect.  The findings suggest that the consideration of metacognitive beliefs, as well as schemas, may be important in understanding non-clinical paranoia. |
| Zamperoni et al. (2022) | USA  Cross-sectional study | 258 Non-Clinical Sample  (198M/60F) Outpatient | 33.62 (12.04) | Childhood Trauma Questionnaire (CTQ) Psychosis Screening Questionnaire (PSQ) Beck Depression Inventory (BDI) Beck Anxiety Inventory (BAI) Brief Core Schema Scales (BCSS) Depression Anxiety Stress Scale (DASS-21) Peters Delusion Inventory (PDI) Locus of Control Scale (LOC) | Results showed that individuals with negative other schemas are more likely to experience unusual thoughts and be distressed and preoccupied by them, whereas negative self-schemas might not have the same effect. Positive schemas did not relate to delusions as measured by the PDI. These findings highlight that negative other schemas may be more critically related to delusional experiences than negative self-schemas.   These results suggest that therapy should include examining and challenging other schemas as part of its therapeutic approach. |

*Key: ARMS, At-risk Mental State; AVH, Auditory Verbal Hallucinations; BD, Bipolar Disorder; BPD, Borderline Personality Disorder; CAARMS, Comprehensive Assessment of At-Risk Mental States; CAMHS, Child and Adolescent Mental Health Services; CHR, Clinical High-risk; D, Depression; DEP, Depression; F, Female; HC, Healthy Controls; M, Male; MDD, Major Depressive Disorder; MH, Mental Health; OCD, Obsessive-compulsive Disorder; PD, Persecutory Delusions; PE, Psychotic Experiences; PLE/PLES, Psychotic-like Experiences; PNS, Persistent Negative Symptoms; PPS, Persistent Positive Symptoms; SB, Siblings; SSD, Schizophrenia-spectrum Disorder; SZ, Schizophrenia; SZA, Schizoaffective Disorder; UES, Unusual Experiences; UHR, Ultra-high risk; VH, Voice Hallucinations.*
